# Phase I studies of AZD1208, a proviral integration Moloney virus kinase inhibitor in solid and haematological cancers

**DOI:** 10.1038/s41416-018-0082-1

**Published:** 2018-05-16

**Authors:** Jorge Cortes, Kenji Tamura, Daniel J. DeAngelo, Johann de Bono, David Lorente, Mark Minden, Geoffrey L. Uy, Hagop Kantarjian, Lisa S. Chen, Varsha Gandhi, Robert Godin, Karen Keating, Kristen McEachern, Karthick Vishwanathan, Janet Elizabeth Pease, Emma Dean

**Affiliations:** 1 Department of Leukemia, Division of Cancer Medicine, The University of Texas MD Anderson Cancer Center, 1901 East Road, Houston, TX 77054 USA; 2 Department of Breast Oncology and Medical Oncology, National Cancer Center Hospital, 5-1-1 Tsukiji, Chuo, Tokyo, 104-0045 Japan; 3 Department of Medical Oncology, Dana-Farber Cancer Institute, 450 Brookline Avenue, Room D-2050, Boston, MA 02215 USA; 4 Prostate Cancer Targeted Therapy Group and Drug Development Unit, Royal Marsden, Downs Road, Sutton, Surrey, SM2 5PT UK; 5 Division of Stem Cell and Developmental Biology, Ontario Cancer Institute, Princess Margaret Hospital, 610 University Avenue, Toronto, ON M5G 2M9 Canada; 6 Department of Medicine, Oncology Division, Washington University School of Medicine, 660 S. Euclid Ave., St. Louis, MO 63110 USA; 7 Department of Experimental Therapeutics, Division of Cancer Medicine, The University of Texas MD Anderson Cancer Center, 1901 East Road, Houston, TX 77054 USA; 8 AstraZeneca, 35 Gatehouse Dr, Waltham, MA 02451 USA; 9 AstraZeneca, 35 Gatehouse Dr, Waltham, MA 02451 USA; 10 AstraZeneca, 310 Cambridge Science Park, Milton Road, Cambridge, CB4 0FZ UK; 11 Clinical Trials Unit, The Christie NHS Foundation Trust, Manchester, M20 4BX UK

**Keywords:** Targeted therapies, Cancer therapy

## Abstract

**Background:**

Proviral integration Moloney virus (PIM) kinases (PIM1, 2 and 3) are overexpressed in several tumour types and contribute to oncogenesis. AZD1208 is a potent ATP-competitive PIM kinase inhibitor investigated in patients with recurrent or refractory acute myeloid leukaemia (AML) or advanced solid tumours.

**Methods:**

Two dose-escalation studies were performed to evaluate the safety and tolerability, and to define the maximum tolerated dose (MTD), of AZD1208 in AML and solid tumours. Secondary objectives were to evaluate the pharmacokinetics, pharmacodynamics (PD) and preliminary efficacy of AZD1208.

**Results:**

Sixty-seven patients received treatment: 32 in the AML study over a 120–900 mg dose range, and 25 in the solid tumour study over a 120–800 mg dose range. Nearly all patients (98.5%) in both studies experienced adverse events, mostly gastrointestinal (92.5%). Dose-limiting toxicities included rash, fatigue and vomiting. AZD1208 was not tolerated at 900 mg, and the protocol-defined MTD was not confirmed. AZD1208 increased CYP3A4 activity after multiple dosing, resulting in increased drug clearance. There were no clinical responses; PD analysis showed biological activity of AZD1208.

**Conclusions:**

Despite the lack of single-agent clinical efficacy with AZD1208, PIM kinase inhibition may hold potential as an anticancer treatment, perhaps in combination with other agents.

## Introduction

Proviral integration Moloney virus (PIM) kinases (PIM1, 2 and 3) are a family of nuclear and cytoplasmic serine (S)/threonine (T) kinases that regulate apoptosis and control cell cycle progression by phosphorylating substrates such as Bcl-2 antagonist of cell death (BAD),^1,2^ p21Cip1/WAF1^3^ and cMyb.^4^

PIM kinases appear overexpressed in several tumour types, contributing to oncogenesis.^5,6^ For example, PIM1 is overexpressed in ~30% of haematopoietic malignancies, particularly in acute myeloid leukaemia (AML) and acute lymphoblastic leukaemia.^7^ PIM1 in T cells induces the formation of lymphoma and increases the rate of lymphoma development in response to Murine Leukaemia Virus.^6^ Furthermore, PIM1 and PIM2 are overexpressed in haematological malignancies^8,9^ and solid tumours.^9,10^

AZD1208 is a potent, ATP-competitive, pan-PIM kinase inhibitor designed to target PIM1, 2 and 3.^11^ It has been investigated in preclinical models of AML and prostate cancer.^12,13^ In AML cell lines, inhibition of cell growth by AZD1208 correlated with PIM1 expression. AZD1208 induced cell cycle arrest and apoptosis, which were accompanied by dose-dependent reductions in levels of phosphorylated BAD, 4E-BP1, p70S6K and S6 proteins.^12^

Here, we report the results of two parallel phase I dose-escalation studies using the PIM kinase inhibitor, AZD1208, which examined the safety, tolerability, pharmacokinetics (PK) and preliminary efficacy of AZD1208 in patients with recurrent or refractory AML or advanced solid tumours.

## Materials and Methods

### Study objectives

Both dose-escalation studies recruiting patients with AML (ClinicalTrials.gov, NCT01489722) and advanced solid malignancies (NCT01588548) were phase I, open-label, multicentre studies designed to identify the maximum tolerated dose (MTD) and evaluate the safety and tolerability of AZD1208 administered orally once daily (QD). Secondary objectives included evaluation of the drug PK and preliminary evidence of efficacy. The AML study also explored pharmacodynamic (PD) biomarkers.

### Patient eligibility

Prior to participation in either study, all patients signed an informed consent document approved by the Institutional Review Board at each site. Both studies were conducted according to the Declaration of Helsinki.

For the AML study, eligible patients were ≥18 years of age with relapsed/refractory AML, with AML secondary to myelodysplastic syndromes or myeloproliferative neoplasm, or with chronic myeloid leukaemia in blast phase. Patients were required to have Eastern Oncology Cooperative Group Performance Status (ECOG PS) 0–2 and be considered likely to complete at least 4 weeks of therapy.

For the solid tumour study, eligible patients were ≥18 years of age and diagnosed with advanced solid tumours—including non-Hodgkin lymphoma—refractory to standard therapies, or for which no standard therapies exist. Eligible patients were required to have ECOG PS 0–1, life expectancy of ≥12 weeks, and ≥1 lesion that could be accurately assessed by Response Evaluation Criteria in Solid Tumours (RECIST) v.1.1 using computed tomography (CT) or magnetic resonance imaging.

Patients were excluded from both studies if they had concomitant uncontrolled diseases, including uncontrolled diabetes, high cholesterol and high white blood cell count (>100,000/mm^3^). Prior allogeneic haematopoietic cell transplantation was allowed, as long as the patients were not still requiring immunosuppression.

### Treatment plan and study design

#### AML dose-escalation study

Conducted at three centres in the USA and one in Canada. Dose escalation followed a conventional 3 + 3 design. The starting dose of AZD1208 was 120 mg QD orally continuously in each 28-day cycle. In the first cohort, dosing between the first and subsequent patients was staggered with a 7-day interval. Dose-escalation decisions were based on the safety and tolerability data from ≥3 evaluable patients (or six if one of the first three patients experienced a dose-limiting toxicity [DLT]).

Evaluable patients must have received ≥75% of the specified AZD1208 dose, or experienced a DLT, during the first 28-day cycle. AZD1208 could be escalated by up to 100% in subsequent cohorts until one DLT was observed, after which succeeding doses were escalated by up to 50%.

In the case of a DLT, the cohort was expanded to six patients. If a DLT occurred in ≥2 patients within a cohort, the dose was determined to be a non-tolerated dose (NTD) and dose escalation was stopped.

Grade 3 or 4 toxicities not attributable to the disease or disease-related processes under investigation, and occurring before the end of Cycle 1, were considered DLTs. The following were also considered DLTs: QTc prolongation (>500 ms; Fridericia’s correction) or an increase of >60 ms from baseline QTc to a QTc value >480 ms, confirmed on repeat 48-h electrocardiogram (ECG); Common Terminology Criteria for Adverse Events (CTCAE) grade 3 or 4 vomiting lasting >24 h, despite suitable antiemetics; grade 5 (death, unless clearly unrelated to therapy [e.g., accidental, due to progressive disease]) or any other toxicity judged to be a DLT by the Safety Review Committee; marrow aplasia continuing for ≥42 days in the absence of leukaemia.

The NTD was identified as the dose at which ≥2 DLTs occurred in a given cohort. The MTD was defined as the highest dose at which <33% of six patients experienced a DLT.

#### Solid tumour dose-escalation study

Conducted at two UK centres and one in Japan. Dose escalation followed a rolling six design.^14^ Patients received a single dose on Day 1 during Cycle 0, followed by a 3-day washout period, after which multiple dosing was initiated. Cycle 1 was a 21-day period from the first dose of multiple dosing.

The dose decisions for a cohort and definition for an evaluable patient were the same as in the AML dose-escalation study. DLTs were defined as: QTc prolongation (>500 ms) or an increase of >60 ms from baseline QTc to a QTc value >480 ms, confirmed on repeat 48-h ECG; haematological toxicity (≥grade 4 present for >4 days [including grade 4 thrombocytopenia, regardless of duration]); anaemia, as defined by haemoglobin <6.5 g/dl (<4.0 mmol/l); febrile neutropenia (including grade 3 neutropenia and temperatures >38.5 °C); grade 3 thrombocytopenia with grade 3 haemorrhagic events; non-haematological toxicity ≥CTCAE grade 3, including diarrhoea, nausea or vomiting persisting for >3 days despite aggressive management; any other toxicity greater than that at baseline, clinically significant and/or unacceptable, not responding to supportive care and resulting in a disruption of the dosing schedule for >14 days.

Administration of AZD1208 began at 120 mg QD, with subsequent dosing levels adjusted based on emerging safety and PD data.

### Assessments

#### Safety

Safety and tolerability were assessed from the time of informed consent until the end of follow-up (defined as 30 and 28 days after study treatment was discontinued for the AML and solid tumour studies, respectively) by evaluation of adverse events (AEs), vital signs, ECGs and laboratory assessments. The CTCAE (version 4.0) was utilised to grade all AE events.

#### PK sampling

The schedules for collection of blood and urine samples for PK analyses in each cohort in both studies are described in Supplementary Table 1. When necessary for clinical response assessment in the AML study, a bone marrow sample for PK analysis was collected at Cycle 1, Day 28.

In the solid tumour study, blood samples for 4β-hydroxycholesterol analysis were collected pre-dose at Cycle 0, Day 1; Cycle 1 (Day 8 and 15); and Cycle 2, Day 15.

The concentration of AZD1208 in plasma and urine was determined by Covance, on behalf of Clinical Bioanalysis Alliance at AstraZeneca R&D, using a bioanalytical method. A volume of 0.05 ml of K_2_EDTA human plasma sample was extracted by liquid–liquid extraction using 0.7 ml of methyl tert-butyl ether. Approximately 100 µl of the supernatant was transferred, evaporated to dryness and reconstituted with 350 µl of 1:1 methanol:water, and a 5 µl injection was made to the high-performance liquid chromatography (HPLC) column. HPLC separations were performed on a Phenomenex LUNA C18 (50 × 2 mm, 5 µm) column using a mobile phase of 0.1% formic acid in water (A) and 0.1% formic acid in acetonitrile (B) at a flow rate of 0.6 ml/min and a column temperature of 40 °C. AZD1208-D5 was used as the internal standard. Detection was performed in a Sciex API4000 mass spectrometer, in a positive electrospray ionisation mode, using multiple reaction monitoring detection (AZD1208: 380.0–>248.1; AZD1208-D5: 385.0–>253.1).

### PD sampling

#### AML dose-escalation study

For AZD1208 PD evaluations, bone marrow aspirates were collected (pretreatment and Cycle 1, Day 1, 2–6 h following administration of AZD1208) and peripheral blood samples were collected (pretreatment on Cycle 1, Day 1; and on-treatment at Cycle 1, Day 1 at 3, 6 and 24 h post-dose; Cycle 1, Day 14 at pre-dose, 3 and 6 h post-dose). Mononuclear cells were isolated, and protein lysates prepared, for subsequent analysis of phosphorylated BAD at S112 by MesoScale Discovery ELISA and phosphorylation of 4E-BP1 at S65 by NanoPro immunoassay [*unpublished data: McEachern* et al. 2018, manuscript in preparation]. Samples were considered evaluable when the baseline biomarker levels were above the background signal (as defined by buffer-only negative control samples) and within the linear range of assay. Additionally, only blood samples containing detectable blasts were considered for analysis.

#### Isolation of primary leukaemia cells for protein profiling

In the AML study, a protein profiling analysis was carried out on primary blasts obtained from peripheral blood of patients (*n* = 6) before and during therapy with AZD1208. Due to time-sensitive aspects of the analysis, only patients from the MD Anderson Cancer Center (MDACC, Houston, TX, USA) were eligible. Whole blood was collected in heparinised tubes. Leukaemia cells were then isolated using Ficoll–Hypaque (specific gravity, 1.086; Life Technologies, Grand Island, NY, USA) density gradient separation, as previously described.^15^ Cell number and mean cell volume were determined using a Coulter Channelyzer (Coulter Electronics, Hialeah, FL, USA).

Mutation analysis was determined for the AML study patients using the fluorescent multiplex polymerase chain reaction and restriction digestion method, followed by capillary electrophoresis at MDACC.

AML blasts isolated from patients during therapy with AZD1208 were harvested and submitted for reverse phase protein array (RPPA) analysis to evaluate protein level changes across a set of 171 antibodies,^16^ graphed using GraphPad Prism software (GraphPad Software, Inc., San Diego, CA, USA).

#### Tumour response

The antileukaemic activity in AML was according to the International Working Group (IWG) response criteria for AML.^17^ Response criteria were modified such that an M-1 marrow was defined as 1% to ≤5% and a partial response as >5% to 25%.^18^

In the solid tumour study, responses were evaluated using RECIST v.1.1. Contrast-enhanced CTs of the chest, abdomen, pelvis and neck were performed at screening (≤28 days before the start of study treatment), every 6 weeks (±1 week) up to 12 weeks, and then every 12 weeks (±1 week) until discontinuation of study treatment or withdrawal of consent, starting from Day 1 of Cycle 1.

### Statistical analysis

AEs were summarised by Medical Dictionary for Regulatory Activities (MedDRA) system organ class, MedDRA preferred term and CTCAE grade. Summary statistics of mean, median, standard deviation, minimum, maximum and number of observations were used.

## Results

### Baseline data and treatment overview

#### AML dose-escalation study

Patient demographics and baseline characteristics were similar in each cohort and are shown in Table 1.

**Table 1:** Patient baseline characteristics in the AML and solid tumour dose-escalation studies

	AML dose-escalation study *n* *=* 32	Solid tumour dose-escalation study *n* *=* 35
Age, years		
Median	65	65
Range	18–89	28–81
Sex		
Male	21 (65.6)	17 (48.6)
Female	11 (34.4)	18 (51.5)
Race		
White	26 (81.3)	19 (54.3)
Asian	3 (9.4)	16 (45.7)
Other	3 (9.4)	0
Extent of disease at baseline		
Locally advanced	NA	13 (37.1)
Metastatic	NA	31 (88.6)
Disease under study		
Refractory AML (primary only)	21 (65.6)	NA
First relapse	3 (9.4)	NA
Second relapse	4 (12.5)	NA
Third or further relapse	4 (12.5)	NA
ECOG PS		
0	6 (18.8)	16 (47.1)
1	21 (65.6)	18 (52.9)
2	5 (15.6)	0
Prior therapy		
Surgery	NA	27 (77.1)
Radiotherapy	2 (6.3)	35 (100)
Chemotherapy, *n* (%), median	32 (100), 4.0	34 (97.1), NA
Immuno-/hormonal therapy	NA	6 (17.1)
Other systemic anticancer therapy^a^	2 (6.3)	0
Stem cell transplant	5 (15.6)	NA
Molecular mutation status		
FLT3		
Detected	3 (9.4)	NA
Not detected	12 (37.5)	NA
Unknown	17 (53.1)	NA
NPM1		
Detected	0	NA
Not detected	12 (37.5)	NA
Unknown	20 (62.5)	NA
Cytogenetics		
Normal	14 (43.8)	NA
t (8:21)	1 (3.1)	NA
Inv 16 or t (16:16)	1 (3.1)	NA
Abnormalities of 5 and/or 7	7 (21.9)	NA
Complex (>3 abnormalities)	8 (25.0)	NA
Other	14 (43.8)	NA

Data are *n* (%) unless otherwise stated.

*AML* acute myeloid leukaemia, *ECOG PS* Eastern Oncology Cooperative Group Performance Status, *FLT3* FMS-like tyrosine kinase 3, *NA* not applicable, *NPM1* nucleophosmin.

^a^Details of ‘other systemic anticancer prior therapy’ are not known

A total of 55 patients were enrolled into the dose-escalation phase of the AML study between 10 February 2012 and 13 May 2014, 32 of whom were assigned to treatment. The other 23 patients were screen failures. All patients assigned to treatment received ≥1 dose of AZD1208 at: 120 mg, *n* *=* 4; 240 mg, *n* *=* 6; 480 mg, *n* *=* 6; 700 mg, *n* *=* 7; and 900 mg, *n* *=* 9. On Day 28 (Cycle 1 completion), 11 patients were receiving AZD1208 (*n* *=* 2 in each of the 120, 480, 700 and 900 mg dose cohorts; 240 mg, *n* *=* 3). Three patients completed Cycle 2 (*n* *=* 1 in each of the 480, 700 and 900 mg dose cohorts). By Day 84, all patients had discontinued treatment.

Among all dose levels, treatment duration ranged from 4–66 days (median range, 15–27 days). One patient in each of the 240 mg (rash), 480 mg (stomatitis) and 700 mg (febrile neutropenia) dose cohorts, and two patients in the 900 mg cohort (one with hypotension, pyrexia and thrombocytopenia; one with rash), had one dose interruption due to AEs. No patients required dose reduction. All 32 eligible patients eventually discontinued study treatment due to a lack of therapeutic response (*n* *=* 19, 59.4%), AEs (*n* *=* 8, 25.0%) and patient’s decision (*n* *=* 5, 15.6%).

#### Solid tumour dose-escalation study

Patient demographics and baseline characteristics were similar in each cohort and are shown in Table 1. Overall, 43 patients were enrolled in the solid tumour study between 17 July 2012 and 14 April 2014, 35 of whom were assigned to treatment (120 mg, *n* = 3; 240 mg, *n* = 7; 360 mg, *n* = 6; 540 mg, *n* = 7; 700 mg, *n* = 6; and 800 mg, *n* = 6).

Twenty-one (60.0%) patients completed the Cycle 1, Day 21 DLT evaluation period (120 mg, *n* *=* 3; 240 mg, *n* *=* 5; 360 mg, *n* *=* 2; 540 mg, *n* *=* 5; 700 mg, *n* *=* 3; and 800 mg, *n* *=* 3). Reasons for not completing the DLT evaluation period were progressive disease (*n* *=* 8, 22.9%), patient decision (*n* *=* 3, 8.6%) and AE (*n* *=* 3, 8.6%).

The median treatment duration was 41 days, but varied considerably (range, 10–357 days). Mean duration was greater for the lower doses (120–540 mg: 78–91 days) than the higher doses (700 mg and 800 mg: 29–44 days). All 35 (100%) patients eventually discontinued study treatment due to disease progression (*n* *=* 23; 65.7%), patient decision to discontinue (*n* *=* 8, 22.9%) or AEs (*n* *=* 4, 11.4%).

### Safety and tolerability

#### AML dose-escalation study

AEs: Overall, 31 patients (96.9%) experienced an AE (Table 2). Gastrointestinal disorders were the most commonly reported AEs (*n* = 28, 87.5%), most frequently nausea (*n* = 15, 46.9%) and diarrhoea (*n* = 14, 43.8%). AEs judged by the investigator to be possibly related to AZD1208 occurred in 22 patients (68.8%), with the most common being nausea (*n* = 12, 37.5%), diarrhoea (*n* = 7, 21.9%), vomiting (*n* = 6, 18.8%) and fatigue (*n* = 6, 18.8%). No clinically significant ECG abnormalities were observed. Seventy-five percent of patients (*n* *=* 24) experienced a grade ≥3 AE, with febrile neutropenia (*n* *=* 9, 28.1%), hypotension (*n* *=* 6, 18.8%) and pneumonia (*n* *=* 5, 15.6%) the most commonly reported.

**Table 2:** All-grade AEs with a frequency of >10% in the AML dose-escalation study (safety analysis set)

MedDRA preferred term	AZD1208 120 mg *n* *=* 4	AZD1208 240 mg *n* *=* 6	AZD1208 480 mg *n* *=* 6	AZD1208 700 mg *n* *=* 7	AZD1208 900 mg *n* *=* 9	Total *N* *=* 32
Patients with any AE	3 (75.0)	6 (100.0)	6 (100.0)	7 (100.0)	9 (100.0)	31 (96.9)
Gastrointestinal						
Nausea	3 (75.0)	4 (66.7)	1 (16.7)	5 (71.4)	2 (22.2)	15 (46.9)
Diarrhoea	1 (25.0)	1 (16.7)	3 (50.0)	5 (71.4)	4 (44.4)	14 (43.8)
Vomiting	2 (50.0)	3 (50.0)	2 (33.3)	2 (28.6)	1 (11.1)	10 (31.3)
Abdominal pain	1 (25.0)	0	1 (16.7)	3 (42.9)	1 (11.1)	6 (18.8)
Stomatitis	1 (25.0)	1 (16.7)	3 (50.0)	0	0	5 (15.6)
Decreased appetite	0	1 (16.7)	0	2 (28.6)	1 (11.1)	4 (12.5)
General disorders						
Fatigue	1 (25.0)	1 (16.7)	4 (66.7)	2 (28.6)	2 (22.2)	10 (31.3)
Oedema peripheral	1 (25.0)	1 (16.7)	3 (50.0)	2 (28.6)	2 (22.2)	9 (28.1)
Asthenia	1 (25.0)	0	1 (16.7)	1 (14.3)	2 (22.2)	5 (15.6)
Chills	0	1 (16.7)	0	1 (14.3)	2 (22.2)	4 (12.5)
Pyrexia	0	0	1 (16.7)	0	3 (33.3)	4 (12.5)
Vascular disorders						
Hypotension	1 (25.0)	2 (33.3)	2 (33.3)	3 (42.9)	2 (22.2)	10 (31.3)
Blood and lymphatic system disorders						
Febrile neutropenia	3 (75.0)	2 (33.3)	0	1 (14.3)	3 (33.3)	9 (28.1)
Respiratory disorders						
Cough	0	1 (16.7)	1 (16.7)	4 (57.1)	2 (22.2)	8 (25.0)
Dyspnoea	0	1 (16.7)	2 (33.3)	2 (28.6)	2 (22.2)	7 (21.9)
Metabolic and nutritional disorders						
Hypocalcaemia	1 (25.0)	1 (16.7)	1 (16.7)	3 (42.9)	1 (11.1)	7 (21.9)
Hypokalaemia	0	0	3 (50.0)	2 (28.6)	2 (22.2)	7 (21.9)
Hypomagnesaemia	1 (25.0)	1 (16.7)	2 (33.3)	2 (28.6)	1 (11.1)	7 (21.9)
Hypophosphataemia	1 (25.0)	0	2 (33.3)	1 (14.3)	2 (22.2)	6 (18.8)
Hyperglycaemia	0	0	2 (33.3)	1 (14.3)	2 (22.2)	5 (15.6)
Dehydration	0	0	0	0	4 (44.4)	4 (12.5)
Infections and infestations						
Pneumonia	1 (25.0)	3 (50.0)	2 (33.3)	0	0	6 (18.8)
Nervous system disorders						
Headache	0	0	3 (50.0)	1 (14.3)	0	4 (12.5)
Skin and subcutaneous disorders						
Rash	0	0	1 (16.7)	1 (14.3)	2 (22.2)	4 (12.5)
Maculopapular rash	0	1 (16.7)	0	2 (28.6)	1 (11.1)	4 (12.5)
Renal and urinary disorders						
Renal failure acute	0	1 (16.7)	2 (33.3)	1 (14.3)	0	4 (12.5)
Eye disorders						
Vision blurred	1 (25.0)	0	3 (50.0)	0	0	4 (12.5)

Data are *n* (%) patients with AEs, sorted in decreasing frequency of preferred term (sorted by total column even when not reported). The number of evaluable patients in each dose cohort was: 120 mg, *n* *=* 3; 240 mg, *n* *=* 3; 480 mg, *n* *=* 3; 700 mg, *n* *=* 4; 900 mg, *n* *=* 3. Data include AEs with an onset date on or after the date of first dose and up to and including 30 days following the date of last dose of study medication. MedDRA version 17.0. was used.

*AE* adverse event, *AML* acute myeloid leukaemia, *MedDRA* Medical Dictionary for Regulatory Activities

Serious AEs (SAEs) were reported in 71.9% (*n* *=* 23) of patients. Febrile neutropenia (*n* *=* 8, 25.0%), hypotension, abdominal pain, maculopapular rash and back pain (each *n* *=* 2, 6.3%) were SAEs reported in >1 patient. Treatment-related SAEs occurred in five (15.6%) patients: two in the 700 mg dose cohort (one each of Guillain–Barré syndrome [GBS] and increased blood creatinine) and three in the 900 mg dose cohort (one febrile neutropenia, two rash).

In total, AZD1208 was discontinued in eight patients (25%), as a result of SAEs and AEs (treatment-related and non-treatment-related): 240 mg, *n* = 1 (bacteremia [SAE] and peristomal ulcer [AE]); 480 mg, *n* = 2 (vomiting [AE] and gingival pain [AE]); 700 mg, *n* = 3 (lung infection, GBS and *Escherichia spp*. sepsis [SAEs]); and 900 mg, *n* = 2 (acute coronary syndrome and rash [SAEs]). These AEs and SAEs were considered treatment-related in four patients: 240 mg, *n* = 1 (peristomal ulcer [AE]); 480 mg, *n* = 1 (vomiting [AE]); 700 mg, *n* = 1 (GBS [SAE]); and 900 mg, *n* = 1 (rash [SAE]).

There were nine deaths during the study, all attributed by the investigators to disease progression.

##### DLTs

DLTs were reported in five patients and occurred between 1–10 days after the start of treatment: 240 mg, *n* = 1 (peristomal ulcer); 480 mg, *n* = 1 (fatigue); 700 mg, *n* = 1 (GBS); 900 mg, *n* = 2 (both rash) (Table 3). One patient with maculopapular rash was rechallenged with AZD1208 at the same dose with no recurrence of the DLT. The other DLTs of rash and GBS resulted in discontinuation of AZD1208.

**Table 3:** DLTs with AZD1208 therapy and treatment decisions in the AML and solid tumour trials

	AZD1208 120 mg	AZD1208 240 mg	AZD1208 360 mg	AZD1208 480 mg	AZD1208 540 mg	AZD1208 700 mg	AZD1208 800 mg	AZD1208 900 mg
*AML study*	*n* *=* 4	*n* *=* 6	NA	*n* *=* 6	NA	*n* *=* 7	NA	*n* *=* 9
DLT, *n*	0	1	—	1	—	1	—	2
Detail, action taken	—	Peristomal ulcer, drug permanently discontinued	—	Fatigue, drug interrupted	—	GBS, drug permanently discontinued	—	Rash, drug permanently discontinued; maculopapular rash, drug permanently discontinued
*Solid tumour study*	*n* *=* 3	*n* *=* 7	*n* *=* 6	NA	*n* *=* 7	*n* *=* 6	*n* *=* 6	NA
DLT, *n*	0	1	0	—	1	0	2	—
Detail, action taken	—	GGT increased, drug interrupted	—	—	Vomiting, drug permanently discontinued	—	Fatigue, drug interrupted; fatigue, drug interrupted	—

*AML* acute myeloid leukaemia, *DLT* dose-limiting toxicity, *GBS* Guillain–Barré syndrome, *GGT* gamma-glutamyltransferase, *NA* not applicable

During the study, no cohort comprised the minimum of six evaluable patients that was required to define MTD (a total of 3/7 patients completed Cycle 1 at 700 mg). MTD was not determined as the dose level below the NTD did not contain the six evaluable patients required to define the MTD of AZD1208.

#### Solid tumour dose-escalation study

##### AEs

All 35 patients reported ≥1 AE (Table 4). Most AEs were gastrointestinal disorders (*n* = 34, 97.1%), the most common being diarrhoea (*n* = 29, 82.9%), nausea (*n* = 26, 74.3%) and vomiting (*n* = 19, 54.3%). All 35 patients had an AE considered to be causally related to AZD1208; diarrhoea (*n* = 24, 68.6%) and nausea (*n* = 23, 65.7%) were the most common.

**Table 4:** All-grade AEs with a frequency of >10% in the solid tumour dose-escalation study (safety analysis set)

MedDRA preferred term	AZD1208 120 mg *n* *=* 3	AZD1208 240 mg *n* *=* 7	AZD1208 360 mg *n* = 6	AZD1208 540 mg *n* = 7	AZD1208 700 mg *n* = 6	AZD1208 800 mg *n* *=* 6	Total *N* *=* 35
Patients with any AE	3 (100.0)	7 (100.0)	6 (100.0)	7 (100.0)	6 (100.0)	6 (100.0)	35 (100.0)
Gastrointestinal disorders							
Diarrhoea	2 (66.7)	5 (71.4)	5 (83.3)	6 (85.7)	5 (83.3)	6 (100.0)	29 (82.9)
Nausea	1 (33.3)	6 (85.7)	3 (50.0)	7 (100.0)	5 (83.3)	4 (66.7)	26 (74.3)
Vomiting	0 (0.0)	6 (85.7)	1 (16.7)	5 (71.4)	4 (66.7)	3 (50.0)	19 (54.3)
Abdominal pain	0 (0.0)	3 (42.9)	2 (33.3)	2 (28.6)	1 (16.7)	0 (0.0)	8 (22.9)
Constipation	0 (0.0)	0 (0.0)	3 (50.0)	0 (0.0)	2 (33.3)	0 (0.0)	5 (14.3)
Blood and lymphatic system disorders							
Anaemia	3 (100.0)	4 (57.1)	2 (33.3)	4 (57.1)	3 (50.0)	2 (33.3)	18 (51.4)
Thrombocytopenia	0 (0.0)	0 (0.0)	2 (33.3)	1 (14.3)	1 (16.7)	1 (16.7)	5 (14.3)
General disorders							
Fatigue	0 (0.0)	2 (28.6)	3 (50.0)	3 (42.9)	4 (66.7)	5 (83.3)	17 (48.6)
Pyrexia	0 (0.0)	1 (14.3)	3 (50.0)	0 (0.0)	2 (33.3)	0 (0.0)	6 (17.1)
Metabolic and nutritional disorders							
Decreased appetite	1 (33.3)	3 (42.9)	2 (33.3)	4 (57.1)	2 (33.3)	3 (50.0)	15 (42.9)
Hypoalbuminaemia	1 (33.3)	2 (28.6)	2 (33.3)	1 (14.3)	3 (50.0)	0 (0.0)	9 (25.7)
Hyperglycaemia	1 (33.3)	0 (0.0)	1 (16.7)	4 (57.1)	0 (0.0)	2 (33.3)	8 (22.9)
Hyponatraemia	0 (0.0)	0 (0.0)	1 (16.7)	3 (42.9)	1 (16.7)	0 (0.0)	5 (14.3)
Hypokalaemia	0 (0.0)	0 (0.0)	0 (0.0)	2 (28.6)	1 (16.7)	1 (16.7)	4 (11.4)
Investigations^a^							
Platelet count decreased	2 (66.7)	2 (28.6)	1 (16.7)	2 (28.6)	1 (16.7)	1 (16.7)	9 (25.7)
White blood cell count decreased	2 (66.7)	3 (42.9)	1 (16.7)	2 (28.6)	1 (16.7)	0 (0.0)	9 (25.7)
GGT increased	0 (0.0)	2 (28.6)	1 (16.7)	2 (28.6)	0 (0.0)	1 (16.7)	6 (17.1)
Skin and subcutaneous tissue disorders							
Dry skin	0 (0.0)	3 (42.9)	1 (16.7)	2 (28.6)	1 (16.7)	0 (0.0)	7 (20.0)
Respiratory, thoracic and mediastinal disorders							
Dyspnoea	0 (0.0)	1 (14.3)	2 (33.3)	1 (14.3)	0 (0.0)	0 (0.0)	4 (11.4)
Psychiatric disorders							
Insomnia	0 (0.0)	1 (14.3)	1 (16.7)	1 (14.3)	0 (0.0)	1 (16.7)	4 (11.4)
Musculoskeletal and connective tissue disorders							
Musculoskeletal chest pain	0 (0.0)	0 (0.0)	2 (33.3)	1 (14.3)	1 (16.7)	0 (0.0)	4 (11.4)

Data are *n* (%) patients with AEs, sorted in decreasing frequency of preferred term (sorted by total column even when not reported). The number of evaluable patients in each dose cohort was: 120 mg, *n* *=* 3; 240 mg, *n* *=* 5; 360 mg, *n* *=* 2; 540 mg, *n* *=* 5; 700 mg, *n* *=* 3; 800 mg, *n* *=* 3. Data include AEs with an onset date on or after the date of first dose and up to and including 30 days following the date of last dose of study medication. MedDRA version 17.0 was used.

*AE* adverse event, *CTCAE* Common Terminology Criteria for Adverse Events, *GGT* gamma-glutamyltransferase, *MedDRA* Medical Dictionary for Regulatory Activities.

^a^Blood cell count decreases according to CTCAE version 4.0

AEs of grade ≥3 were observed in 16 patients (45.7%). Four patients (11.4%) discontinued the study due to an AE: pneumonitis, *n* *=* 2; vomiting, *n* *=* 1; maculopapular rash, *n* *=* 1. Sixteen patients (45.7%) required dose interruptions because of AEs: 240 mg, *n* *=* 2; 360 mg, *n* *=* 3; 540 mg, *n* *=* 6; 700 mg, *n* *=* 3; 800 mg, *n* *=* 2. One patient in the 800 mg cohort required dose reduction because of AEs.

AEs of CTCAE grade ≥3 that occurred in ≥2 patients included: fatigue (*n* *=* 4 [11.4%]), gamma-glutamyltransferase (GGT) increase (*n* *=* 3 [8.6%]), abdominal pain (*n* *=* 2 [5.7%]) and anaemia (*n* *=* 2 [5.7%]). CTCAE grade ≥3 AEs causally related to AZD1208 occurred in 10 patients (28.6%): 240 mg, *n* *=* 3 (anaemia, alanine transaminase increased, GGT increased); 360 mg, *n* *=* 1 (lymphocyte decreased); 540 mg, *n* *=* 2 (vomiting, fatigue, lethargy); 700 mg, *n* *=* 1 (nausea, fatigue); 800 mg, *n* *=* 3 (fatigue, hyperglycaemia).

Eight patients (22.9%) experienced ≥1 SAE, none of which led to discontinuation of AZD1208. SAEs that occurred in ≥2 patients were dyspnoea (*n* *=* 2, 5.7%) and vomiting (*n* *=* 2, 5.7%). One patient (2.9%) reported three treatment-related SAEs (vomiting, fatigue, general physical health deterioration). One patient (2.9%) died as a result of an AE (general deterioration of physical health) that was not considered to be AZD1208-related by the investigator.

No clinically significant treatment-related changes in haematology, clinical chemistry, vital signs, ECG or physical findings were detected in any patient.

Four patients experienced DLTs: grade 3 fatigue (800 mg, *n* = 2), grade 3 GGT increase (240 mg, *n* = 1) and grade 3 vomiting (540 mg, *n* = 1) (Table 3).

During the study, the 800 mg dose was not expanded to the six evaluable patients required to define MTD. As no dosing cohort met these criteria, the MTD of AZD1208 could not be determined.

### PK

#### AML dose-escalation study

The absorption of AZD1208 after a single dose was rapid, with median time to maximum plasma concentration (T_max_) of ~3 h and concentrations remaining high until 24 h (Supplementary Table 2 and Supplementary Figure 1A). A statistical power analysis demonstrated that following a single dose, the maximum AZD1208 plasma concentration (C_max_) (Supplementary Figure 2) and area under plasma concentration–time curve (AUC_0–t_) (Supplementary Figure 3) generally increased in proportion to the administered dose across cohorts (range, 120–900 mg).

Renal clearance was low across all cohorts, with <1% of the administered dose eliminated unchanged in urine within 24 h. Absorption of AZD1208 was likewise rapid after multiple doses, but highly variable, with up to 10-fold differences in AZD1208 concentrations between individuals even within the same cohort (Supplementary Table 2 and Supplementary Figure 1B).

Across the 120–900 mg doses, 25% (4/16) of patients had significantly lower exposure at steady state compared with the first dose of AZD1208, and the percentage of patients with a lower accumulation ratio increased with increasing dose (0% at 240 mg to 67% at 900 mg). However, 44% (7/16) of patients did show a marked accumulation (>3-fold) across the doses. The number of patients with higher accumulation decreased with increasing doses, and exposure was highly variable, with one patient at 480 mg having an accumulation ratio of 3.6 and another a ratio of 0.36.

In general, both AUC over dosing interval (AUC_tau_) and C_max_ decreased with increasing doses. Power and ANOVA models revealed that whereas C_max_ and AUC_0–24_ were dose proportional on Cycle 1, Day 1, both C_max_ and AUC_0–24_ were less than dose proportional following multiple doses of AZD1208.

#### Solid tumour dose-escalation study

**Fig. 1:**
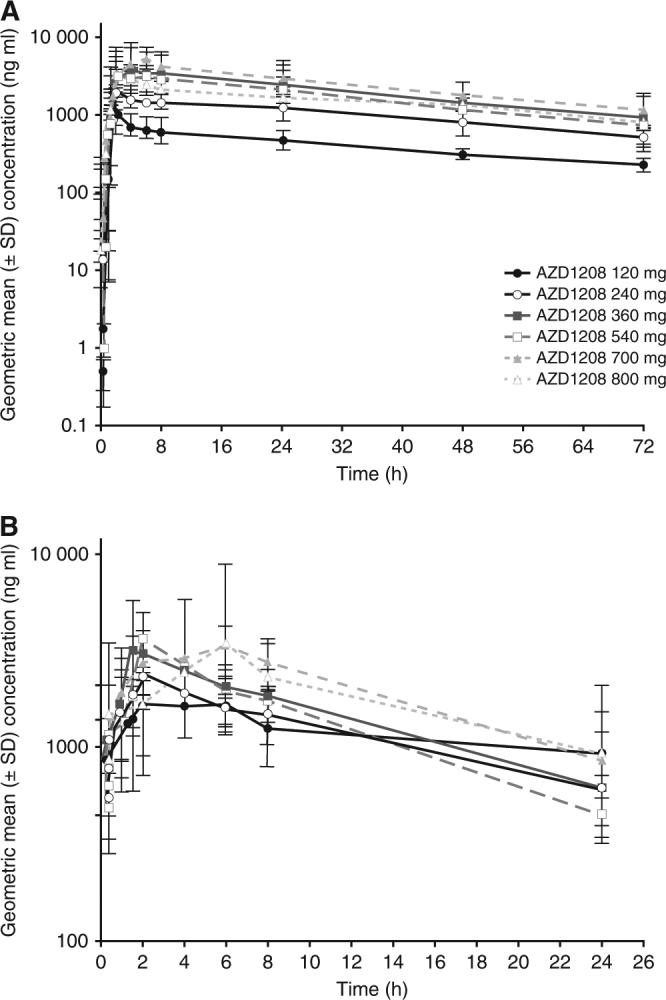
Solid tumour dose-escalation study geometric mean plasma concentration (semi-log scales ± SD) of AZD1208 *vs* time by **a** single dose for Cycle 0, Day 1, and **b** multiple doses for Cycle 1, Day 15. *SD*, standard deviation

After a single dose, the systemic exposure of AZD1208 (AUC and C_max_) was variable but largely proportional with doses up to 700 mg, and less than proportional from 700–800 mg (Fig. 1a and Supplementary Table 3). After multiple dosing, exposure at all doses was similar, with no increase in exposure with increasing doses (Fig. 1b and Supplementary Table 3). Individual and geometric mean values for C_max_ and AUC are presented in Supplementary Figures 2 and 3, respectively.

The mean half-life following a single dose was determined to be 37.2 h (min/max: 18.9 h/103 h). The elimination half-life after multiple dosing could not be accurately determined.

Absorption following single dosing was moderate and variable, with median T_max_ achieved by ~5 h (range, 1.5–25 h); distribution was moderate, and clearance low. In comparison, after multiple dosing, the absorption was moderate, with median T_max_ of ~4 h (range, 1.5–6 h), and clearance increased with increasing dose.

After a single dose, ~1% of the AZD1208 dose was eliminated in urine over 72 h. After multiple dosing, on Day 15 0.5% of the AZD1208 dose was observed in urine. Therefore, renal clearance was negligible.

The activity of CYP3A4 was assessed by analysing 4-β-hydroxycholesterol levels in samples collected from five patients at AZD1208 doses of 700 or 800 mg. At both doses, the levels of 4-β-hydroxycholesterol increased ~4-fold in all patients on Day 15, compared with Day 1 (Supplementary Table 4), indicating that CYP3A4 activity was induced by AZD1208.

### PD

**Fig. 2:**
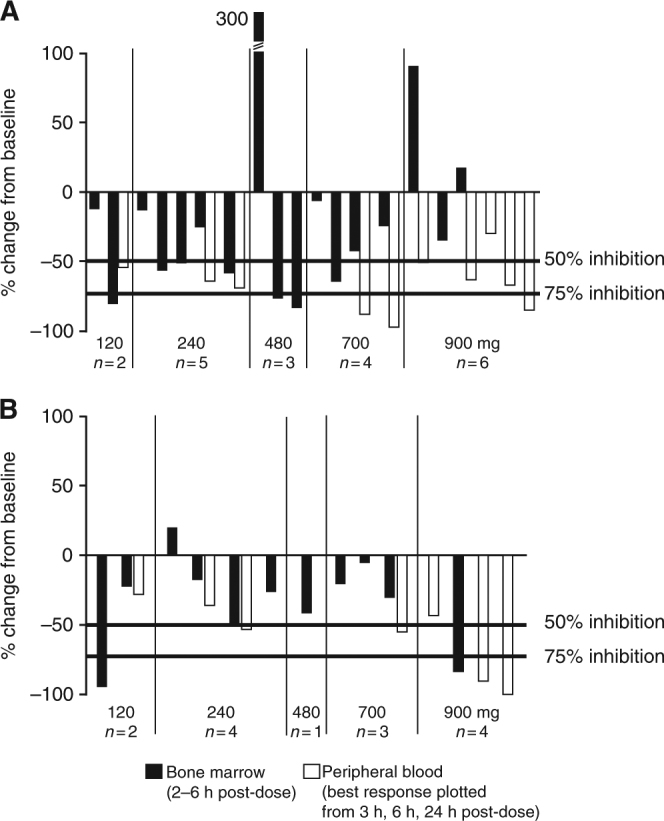
PD response to AZD1208 treatment in the AML dose-escalation study. The percentage of change from baseline in the reduction of (**a**) pBAD S112, and (**b**) p4E-BP1 S65 in bone marrow and peripheral blood samples collected following a single dose of AZD1208 is shown. Only the maximal PD response measured on Day 1 for peripheral blood samples is shown. Each column represents an individual patient, with adjacent columns corresponding with matched marrow and blood samples. *AML*, acute myeloid leukaemia; *PD*, pharmacodynamics

Reductions in pBAD S112 were observed in 7/17 patients with evaluable bone marrow samples in the AML study (Fig. 2a). Reductions in p4E-BP1 S65 were also observed in 3/11 patients with evaluable bone marrow samples (Fig. 2b). However, the small number of patients and variability in PD response in these data must be noted.

Reductions of pBAD S112 in peripheral blood were more frequently observed, with 9/10 patients across all dose levels demonstrating ≥50% reduction from baseline (Fig. 2a). Decreases in p4E-BP1 S65 were seen in the peripheral blood samples from 4/7 patients, with the greatest reductions observed at the highest dose level of 900 mg (Fig. 2b). No correlation between dose, exposure and biomarker effect was identified in these samples.

### AML study—RPPA protein profiling in AML blasts

RPPA analysis of 171 proteins was performed on samples from six patients (Supplementary Figure 1A). Based on the PD analyses using MesoScale (Meso Scale Diagnostics, LLC, MD, USA) and NanoPro (ProteinSimple, CA, USA), and on previous preclinical investigations in AML cell lines,^19^ five phosphoproteins were selected for analysis. Consistent with Fig. 2, there was heterogeneity in the protein level changes of phosphorylated 4E-BP1 S65 and BAD S112, measured using RPPA analysis (Supplementary Figures 4A and 4B). Reductions were seen in 4E-BP1 S65 in three patients (Supplementary Figure 4A) and in BAD S112 in four patients (Supplementary Figure 4B). However, the small number of patients and variability in response in these data must be noted. Similarly, there was variability in the effects on phosphorylated 4E-BP1 T37/46, PRAS40 T246 and mTOR S2448 levels following AZD1208 treatment (Supplementary Figures 5A–5D).

### Efficacy

#### AML dose-escalation study

There were no clinical responses according to IWG criteria (Supplementary Table 5). A reduction in circulating blasts occurred in several patients in the 120, 240 and 480 mg dose cohorts. Only five patients who displayed decreases in circulating blasts had evaluable biomarker samples, three of whom showed a robust decrease in phosphoprotein levels. However, several patients had reduced phosphoprotein levels without a reduction in blasts. Resistant disease was the most common treatment failure reason.

#### Solid tumour dose-escalation study

The best objective response, as assessed by RECIST, was stable disease for ≥6 weeks (*n* = 13) and progression (*n* = 14), with five patients non-evaluable for response assessment (Supplementary Table 5). Patients were not evaluable because they had stable disease response for <6 weeks (*n* = 3) or incomplete post-baseline assessments (*n* = 2). The objective response rate for the study was 0%. At Week 12, only five patients were evaluable for tumour response assessments: four patients had stable disease (120 mg, *n* = 1; 240 mg, *n* = 2 and 540 mg, *n* = 1); one had progressive disease (240 mg). Of note, one patient who received treatment in the solid tumour dose-escalation study had prostate cancer, and experienced a considerable reduction in prostate-specific antigen (PSA) levels.

## Discussion

In the first-in-human, dose-escalation study in patients with heavily pretreated AML, AZD1208 was generally tolerated up to doses of 700 mg, but not tolerated at the highest 900 mg dose. PK data from this study suggest that AZD1208 absorption profiles for patients who experienced DLTs were in the same range as those of patients who did not have a DLT. It is unlikely that dose interruptions contributed to any variability in repeat dosing PK as the majority of the PK sampling timepoints occurred early in the study (first 15 days of treatment in Cycle 1). In the first 15 days of treatment there were minimal to no dose interruptions, and those patients who had dose interruptions were not included in the PK statistical analysis and hence did not contribute to the variability. In the solid tumour dose-escalation study, AZD1208 was tolerated as monotherapy at doses up to 700 mg QD, but not tolerated at 800 mg QD. The MTD was not confirmed in either study. It is possible that the dose below 900 mg was the MTD; however, as we did not enrol the six patients required by the protocol, this cannot be confirmed.

Pan-PIM inhibition with AZD1208 appeared to be generally tolerated in both studies, with the most common AEs affecting the gastrointestinal tract. The patient in the AML study with GBS had a history of *E. coli* bacteremia a few weeks before starting treatment with AZD1208, and concurrent diseases including polyarthritis and gout. Previous anticancer therapies included ruxolitinib, vidaza, vosaroxin, decitabine, fludarabine and cytarabine. In light of this history, it was concluded that these factors may have contributed to the development of GBS.

Another pan-PIM kinase inhibitor, LGH447, is currently in clinical development and shows a manageable safety profile (ClinicalTrials.gov, NCT01456689).^20^ Among the 54 patients who received LGH447 as monotherapy, there were eight DLTs (thrombocytopenia, *n* *=* 4; fatigue, *n* *=* 2; hypophosphatemia, *n* *=* 1; vasovagal syncope, *n* *=* 1), but most AEs were grade 1 or 2.^20^ While pan-inhibition of PIM kinases with AZD1208 and LGH447 appears to be tolerable, development of the pan-PIM kinase inhibitor SGI-1776 in refractory prostate cancer and relapsed/refractory non-Hodgkin lymphoma was discontinued because of QTc prolongation (ClinicalTrials.gov, NCT00848601).

The lack of clinical responses in our AML and solid tumour dose-escalation studies suggests that targeting the PIM pathway with monotherapy may be insufficient to impact refractory AML or advanced solid malignancies. However, PD analysis of AZD1208 activity showed that, in a subset of patients, AZD1208 treatment resulted in a reduction in the phosphorylation of PIM targets, providing evidence for the biological activity of AZD1208 in patients with refractory AML.

Pan-PIM inhibition has demonstrated therapeutic efficacy in a phase I study of LGH447 in 54 patients with relapsed/refractory multiple myeloma (RRMM).^20^ The overall response rate was 10.4%, with a minor response or greater in 20.8% and stable disease or greater in 68.8%.^20^ Therefore, identifying the tumour types that may be more sensitive to PIM inhibition will be important for future PIM inhibitor trials. For instance, PIM overexpression has been strongly associated with prostate cancer;^21,22^ indeed, one patient who received treatment in the solid tumour dose-escalation study had prostate cancer, and experienced a considerable reduction in PSA levels. In MYC-driven prostate cancer models, AZD1208 significantly decreased tumour growth, an effect that was accompanied by decreased cellular proliferation and increased rates of apoptosis. AZD1208 treatment also sensitised the prostate tumours to radiation.^13^

PIM kinase inhibitors may have the potential to be used in combination with other therapies. For example, a phase Ib/II trial of LGH447 in combination with the PI3K inhibitor, BYL719, is underway in patients with RRMM (ClinicalTrials.gov, NCT02144038). In preclinical tumour models, PIM kinase inhibitors also have the ability to sensitise cancer cells to radiotherapy and chemotherapy.^23,24^ For example, in a mouse xenograft model of non-small-cell lung cancer, PIM inhibition sensitised cancer cells to radiation^23^ and PIM antagonism in prostate cancer cells sensitised the cells to the chemotherapeutic gemcitabine.^24^ Thus, PIM kinase inhibitors may be important in contexts where PIM kinases are acting with other therapeutic targets to drive oncogenic progression.

Preclinical studies of AML suggest that mTOR pathway signalling and suppression of protein translation may play a part in the mechanism of action of AZD1208.^19^ Consistent with these studies, significant reductions in 4EBP1 S65 were seen in a subset of patients while more modest decreases in 4E-BP1 T37/46 and mTOR S2448 were also noted. Given the lack of clinical efficacy in the AML trial, it is not clear to what extent effects on protein translation contribute to PIM kinase activity in AML.

Based on the half-life of AZD1208 after a single dose, it was predicted that multiple dosing would lead to accumulation of AZD1208. However, exposure decreased with increasing doses and with duration of dosing, indicating a possible change in clearance. Increased levels of 4-β-hydroxycholesterol following multiple dosing of AZD1208 confirmed that the increase in clearance was due to induction of CYP3A4 enzymatic activity.

In conclusion, AZD1208 was generally tolerated in patients with heavily pretreated AML and advanced solid malignancies in two dose-escalation studies. AZD1208 increased CYP3A4 activity after multiple dosing, resulting in increased drug clearance. There was no clear evidence of antitumour activity with AZD1208 monotherapy, and the MTD was not established. Considering the challenges in managing this potent increase in CYP3A4 activity, in addition to the lack of observed responses in the clinical setting, the development of AZD1208 was terminated. Still, PIM kinase inhibition may be a relevant anticancer strategy, potentially in combination with other agents.

## Electronic supplementary material

Study 1 and 5 supplementary appendix

Supplementary Figure 1

Supplementary Figure 2

Supplementary Figure 3

Supplementary Figure 4

Supplementary Figure 5

## References

[1] Aho TL (2004). Pim-1 kinase promotes inactivation of the pro-apoptotic bad protein by phosphorylating it on the Ser112 gatekeeper site. FEBS Lett..

[2] Yan B (2003). The PIM-2 kinase phosphorylates BAD on serine 112 and reverses BAD-induced cell death. J. Biol. Chem..

[3] Wang Z (2002). Phosphorylation of the cell cycle inhibitor p21Cip1/WAF1 by Pim-1 kinase. Biochim Biophys. Acta.

[4] Winn LM, Lei W, Ness SA (2003). Pim-1 phosphorylates the DNA binding domain of c-Myb. Cell Cycle.

[5] Cuypers HT (1984). Murine leukemia virus-induced T-cell lymphomagenesis: integration of proviruses in a distinct chromosomal region. Cell.

[6] van Lohuizen M (1989). Predisposition to lymphomagenesis in pim-1 transgenic mice: cooperation with c-myc and N-myc in murine leukemia virus-induced tumors. Cell.

[7] Amson R (1989). The human protooncogene product p33pim is expressed during fetal hematopoiesis and in diverse leukemias. Proc. Natl Acad. Sci. USA.

[8] Asano J (2011). The serine/threonine kinase Pim-2 is a novel anti-apoptotic mediator in myeloma cells. Leukemia.

[9] Cohen AM (2004). Increased expression of the hPim-2 gene in human chronic lymphocytic leukemia and non-Hodgkin lymphoma. Leuk. Lymphoma.

[10] Poulsen CB (2005). Microarray-based classification of diffuse large B-cell lymphoma. Eur. J. Haematol..

[11] Dakin LA (2012). Discovery of novel benzylidene-1,3-thiazolidine-2,4-diones as potent and selective inhibitors of the PIM-1, PIM-2, and PIM-3 protein kinases. Bioorg. Med Chem. Lett..

[12] Keeton EK (2014). AZD1208, a potent and selective pan-Pim kinase inhibitor, demonstrates efficacy in preclinical models of acute myeloid leukemia. Blood.

[13] Kirschner AN (2014). PIM kinase inhibitor AZD1208 for treatment of MYC-driven prostate cancer. J. Natl. Cancer Inst..

[14] Skolnik JM, Barrett JS, Jayaraman B, Patel D, Adamson PC (2008). Shortening the timeline of pediatric phase I trials: the rolling six design. J. Clin. Oncol..

[15] Chen LS, Keating MJ, Gandhi V (2014). Blood collection methods affect cellular protein integrity: implications for clinical trial biomarkers and ZAP-70 in CLL. Blood.

[16] The University of Texas MD Anderson Cancer Center. Functional Proteomics RPPA Core Facility. www.mdanderson.org/research/research-resources/core-facilities/functional-proteomics-rppa-core.html (2017).

[17] Cheson BD (2003). Revised recommendations of the international working group for diagnosis, standardization of response criteria, treatment outcomes, and reporting standards for therapeutic trials in acute myeloid leukemia. J. Clin. Oncol..

[18] Kantarjian H (2003). Phase 2 clinical and pharmacologic study of clofarabine in patients with refractory or relapsed acute leukemia. Blood.

[19] Chen LS, Yang JY, Liang H, Cortes JE, Gandhi V (2016). Protein profiling identifies mTOR pathway modulation and cytostatic effects of Pim kinase inhibitor, AZD1208, in acute myeloid leukemia. Leuk. Lymphoma.

[20] Raab MS, et al. Phase 1 study update of the novel Pan–Pim kinase inhibitor LGH447 in patients with relapsed/refractory multiple myeloma. *Blood.* **124**, abstr. 301 (2014).

[21] Nawijn MC, Alendar A, Berns A (2011). For better or for worse: the role of Pim oncogenes in tumorigenesis. Nat. Rev. Cancer.

[22] Shah N (2008). Potential roles for the PIM1 kinase in human cancer—a molecular and therapeutic appraisal. Eur. J. Cancer.

[23] Kim W (2013). PIM1 kinase inhibitors induce radiosensitization in non-small cell lung cancer cells. Pharmacol. Res.

[24] Xu D (2013). Inhibition of oncogenic Pim-3 kinase modulates transformed growth and chemosensitizes pancreatic cancer cells to gemcitabine. Cancer Biol. Ther..

