# Supplementary material for: Phase I studies of AZD1208, a proviral integration Moloney virus kinase inhibitor in solid and haematological cancers
Source: Br J Cancer. 2018 May 16;118(11):1425–33. doi: 10.1038/s41416-018-0082-1 (PMC5988656; doi:10.1038/s41416-018-0082-1)

Phase I studies of AZD1208, a proviral integration Moloney virus kinase inhibitor in solid and haematological cancers

# Supplementary Information

| **Supplementary Table 1: Sampling schedule for pharmacokinetic analysis of AZD1208 in the AML and solid tumour trials** | | | |
| --- | --- | --- | --- |
| **Blood sample** | | **Urine sample** | |
| **AML dose-escalation study** | | | |
| **Cycle/Day** | **Time** | **Cycle/Day** | **Time** |
| 1/1 | 0 h pre-dose | 1/1 | Pre-dose |
| 1/1 | 0.5 h post-dose | 1/1 | 0–24 h |
| 1/1 | 1 h post-dose |  |  |
| 1/1 | 1.5 h post-dose |  |  |
| 1/1 | 2 h post-dose |  |  |
| 1/1 | 3 h post-dose |  |  |
| 1/1 | 6 h post-dose |  |  |
| 1/1 | 8 h post-dose |  |  |
| 1/1 | 24 h post-dose |  |  |
| 1/7 | 0 h pre-dose |  |  |
| 1/14 | 0 h pre-dose | 1/14 | 0–24 h |
| 1/14 | 0.5 h post-dose |  |  |
| 1/14 | 1 h post-dose |  |  |
| 1/14 | 1.5 h post-dose |  |  |
| 1/14 | 2 h post-dose |  |  |
| 1/14 | 3 h post-dose |  |  |
| 1/14 | 6 h post-dose |  |  |
| 1/14 | 8 h post-dose |  |  |
| 1/14 | 24 h post-dose |  |  |
| 1/28 | 2 h post-dose |  |  |
| **Solid tumour dose-escalation study** | | | |
| **Cycle/Day** | **Time** | **Cycle/Day** | **Time** |
| 0/1 | 0 h pre-dose | 0/1 | Pre-dose |
| 0/1 | 0.5 h post-dose | 0/1–2 | 0–24 h |
| 0/1 | 1 h post-dose |  |  |
| 0/1 | 2 h post-dose |  |  |
| 0/1 | 4 h post-dose |  |  |
| 0/1 | 6 h post-dose |  |  |
| 0/1 | 8 h dose-dose |  |  |
| 0/2 | 24 h post-dose | 0/2–3 | 24–48 h |
| 0/3 | 48 h post-dose | 0/3–4 | 48–72 h |
| 0/4 | 72 h post-dose |  |  |
| 1/2 | 0 h pre-dose |  |  |
| 1/5 | 0 h pre-dose |  |  |
| 1/8 | 0 h pre-dose |  |  |
| 1/8 | 2–6 h post-dose |  |  |
| 1/15 | 0 h pre-dose | 1/15–16 | 0–24 h |
| 1/15 | 0.5 h post-dose |  |  |
| 1/15 | 1 h post-dose |  |  |
| 1/15 | 2 h post-dose |  |  |
| 1/15 | 4 h post-dose |  |  |
| 1/15 | 6 h post-dose |  |  |
| 1/15 | 8 h post-dose |  |  |
| 1/16 | 24 h post-dose |  |  |
| 2/1 | 0 h pre-dose |  |  |
| 2/8 | 0 h pre-dose |  |  |
| 2/8 | 0.5 h post-dose |  |  |
| 2/8 | 1 h post-dose |  |  |
| 2/8 | 2 h post-dose |  |  |
| 2/8 | 4 h post-dose |  |  |
| 2/8 | 6 h post-dose |  |  |
| 2/8 | 8 h post-dose |  |  |
| 2/15 | 0 h pre-dose |  |  |
| 3+/1 | 0 h pre-dose |  |  |
| Abbreviation: AML = acute myeloid leukaemia | | | |

.

| **Supplementary Table 2: Summary of AZD1208 plasma concentrations (pharmacokinetic analysis set) in the AML trial** | | | | | |
| --- | --- | --- | --- | --- | --- |
|  | AZD1208  120 mg *n=*3 | AZD1208  240 mg *n=*6 | AZD1208  480 mg *n=*6 | AZD1208  700 mg *n=*7 | AZD1208  900 mg *n=*9 |
| **Cycle 1, Day 1** | | | | | |
| **T_max_, h** |  |  |  |  |  |
| Median | 2.42 | 2.20 | 3.17 | 3.05 | 3.20 |
| Min, max | 2.00, 3.08 | 1.67, 3.08 | 2.00, 23.83 | 1.53, 8.00 | 2.97, 6.00 |
| **G_mean_ plasma concentration of AZD1208, ng ml (± SD)** |  |  |  |  |  |
| Pre-dose | NQ | 76.9 (NC) | NQ | NQ | NQ |
| Post-dose, h |  |  |  |  |  |
| 0.5 | 85.8 (40.1, 183.9) | 158.3 (14.4, 1740.3) | 124.3 (67.6, 228.6) | 386.8 (93.3, 1603.6) | 251.5 (90.7, 697.2) |
| 1 | 321.3 (187.8, 549.9) | 540.4 (133.8, 2182.4) | 573.0 (248.3, 1322.0) | 883.8 (351.6, 2221.7) | 921.4 (373.1, 2275.6) |
| 1.5 | 774.5 (422.1, 1421.3) | 1098.6 (620.9, 1943.7) | 1243.2 (485.9, 3181.1) | 1849.6 (783.5, 4366.4) | 2106.0 (860.5, 5154.2) |
| 2 | 779.9 (508.9, 1195.3) | 1388.6 (1074.3, 1744.9) | 1852.3 (781.9, 4387.6) | 2640.6 (1438.6, 4847.0) | 3553.2 (2352.6, 5366.6) |
| 3 | 715.6 (415.5, 1232.6) | 1299.5 (967.1, 1746.2) | 2865.4 (1884.8, 4356.2) | 3112.3 (1814.6, 5337.9) | 3574.9 (1521.4, 8400.4) |
| 6 | 546.6 (356.7, 837.6) | 1004.2 (822.5, 1226.0) | 2642.0 (1774.2, 3934.4) | 2846.5 (2115.2, 3830.6) | 4252.0 (2883.8, 6269.4) |
| 8 | 481.6 (308.4, 752.3) | 950.3 (825.3, 1094.2) | 2249.0 (1520.2, 3327.1) | 3284.7 (2253.3, 4788.2) | 3882.9 (2438.1, 6184.0) |
| 24 | 199.0 (75.6, 524.0) | 844.5 (598.4, 1191.8) | 2169.6 (1161.6, 4052.3) | 2395.4 (1664.2, 5572.3) | 3045.2 (1664.2, 5572.3) |
| **Cycle 1, Day 14** | | | | | |
| **G_mean_ plasma concentration of AZD1208, ng ml (± SD)** |  |  |  |  |  |
| Pre-dose | 1163.1 (335.5, 4032.0) | 2106.9 (693.2, 6845.2) | 2302.9 (541.6, 9792.3) | 2513.6 (1057.2, 5976.4) | 463.1 (444.2, 482.7) |
| Post-dose, h |  |  |  |  |  |
| 0.5 | 1758.9 (970.3, 3188.5) | 2365.9 (976.3, 6694.0) | 2887.4 (322.6, 25 841.7) | 2029.2 (863.1, 4770.5) | 640.6 (926.0, 955.4) |
| 1 | 2050.2 (1514.6, 2775.2) | 2556.4 (4058.6, 6516.6) | 4956.2 (1164.7, 21 090.8) | 2013.8 (1004.8, 4035.8) | 1952.5 (1166.1, 3269.1) |
| 1.5 | 2375.1 (1849.1, 3050.7) | 5142.8 (4058.6, 6516.6) | 5384.2 (2123.1, 13 654.8) | 3963.7 (2236.9, 7023.5) | 2747.1 (1719.7, 4388.3) |
| 2 | 2216.3 (1388.5, 3537.7) | 6153.8 (5277.1, 7175.8) | 5818.8 (2320.7, 14 589.5) | 4388.1 (2945.6, 6536.9) | 3107.5 (1803.0, 5356.1) |
| 3 | 2052.0 (1181.0, 3565.2) | 3687.8 (1657.8, 8203.5) | 5515.9 (1885.1, 16 139.7) | 5521.1 (2560.5, 11 905.2) | 3179.0 (1505.2, 6714.4) |
| 6 | 1761.5 (1019.2, 3044.5) | 3196.3 (1504.2, 6791.5) | 5420.2 (1806.5, 16 262.9) | 4452.1 (2126.6, 9320.8) | 2320.5 (896.7, 6004.8) |
| 8 | 1700.4 (974.4, 2967.5) | 2735.5 (1203.4, 6218.4) | 5248.1 (1804.2, 15 265.9) | 4064.8 (2043.6, 8085.1) | 2608.4 (2223.6, 3059.7) |
| 24 | 1248.3 (485.6, 3208.8) | 1818.9 (580.7, 5697.5) | 3154.5 (406.4, 24 485.6) | 2302.0 (643.6, 8233.0) | 593.0 (174.0, 2021.0) |
| Abbreviations: AML = acute myeloid leukaemia; G_mean_ = geometric mean; NC = not calculable; NQ = non-quantifiable (below limit of quantification); SD = standard deviation; T_max_ = median time to maximum plasma concentration | | | | | |

| **Supplementary Table 3: Summary of plasma concentration (ng ml) following single doses of AZD1208 (pharmacokinetic analysis set) in the solid tumour trial** | | | | | | |
| --- | --- | --- | --- | --- | --- | --- |
|  | **AZD1208  120 mg *n=*3** | **AZD1208  240 mg *n=*7** | **AZD1208  360 mg *n=*6** | **AZD1208  540 mg *n=*7** | **AZD1208  700 mg *n=*6** | **AZD1208  800 mg *n=*6** |
| **Cycle 0, Day 1** | | | | | | |
| **Time after dose, h** |  |  |  |  |  |  |
| Pre-dose | *n=*3 NC | *n=*7 NC | *n=*6 NC | *n=*7 NC | *n=*6 NC | *n=*2 NC |
| Post-dose, h |  |  |  |  |  |  |
| 0.25 | *n=*3 1.793 | *n=*7 12.83 | *n=*6 29.51 | *n=*7 26.29 | – | – |
| 0.5 | *n=*3 20.28 | *n=*7 257.5 | *n=*6 343.5 | *n=*7 200.2 | *n=*6 139.2 | *n=*6 222.3 |
| 1 | *n=*3 156.2 | *n=*7 668.4 | *n=*5 1424 | *n=*7 1053 | *n=*6 691.7 | *n=*6 925.6 |
| 1.5 | *n=*3 696.5 | *n=*7 1317 | *n=*5 2509 | *n=*7 2747 | – | – |
| 2 | *n=*3 1064 | *n=*7 1906 | *n=*6 3412 | *n=*7 4049 | *n=*6 3514 | *n=*6 2929 |
| 4 | *n=*3 720.6 | *n=*6 1531 | *n=*6 3157 | *n=*7 3160 | *n=*6 4581 | *n=*6 3844 |
| 6 | *n=*3 681 | *n=*7 1408 | *n=*6 2501 | *n=*7 3190 | *n=*6 5091 | *n=*6 2677 |
| 8 | *n=*3 597.8 | *n=*7 1456 | *n=*6 2196 | *n=*7 2957 | *n=*6 4256 | *n=*6 3463 |
| 24 | *n=*3 459.4 | *n=*7 1157 | *n=*6 1649 | *n=*7 2111 | *n=*6 2957 | *n=*6 2466 |
| 48 | *n=*3 308.4 | *n=*7 773.2 | *n=*6 1361 | *n=*7 1168 | *n=*6 1717 | *n=*6 1377 |
| 72 | *n=*3 221.4 | *n=*7 501.1 | *n=*6 912.2 | *n=*7 710.8 | *n=*6 1141 | *n=*6 798.7 |
| **Cycle 1, Day 15** | | | | | | |
| Pre-dose | *n=*3 903.1 | *n=*6 560.9 | *n=*3 602.5 | *n=*4 266.6 | *n=*3 819 | *n=*2 1099 |
| Post-dose, h |  |  |  |  |  |  |
| 0.25 | *n=*3 963.7 | *n=*6 606.8 | *n=*2 562.8 | *n=*3 496.8 | – | – |
| 0.5 | *n=*3 1109 | *n=*6 881.5 | *n=*3 884.4 | *n=*3 774 | *n=*4 1042 | *n=*2 1420 |
| 1 | *n=*3 1319 | *n=*6 1316 | *n=*3 1983 | *n=*3 1691 | *n=*4 1320 | *n=*2 1783 |
| 1.5 | *n=*3 1367 | *n=*6 1843 | *n=*3 3212 | *n=*3 3302 | – | – |
| 2 | *n=*3 1681 | *n=*6 2340 | *n=*3 3106 | *n=*3 3646 | *n=*4 2802 | *n=*2 1705 |
| 4 | *n=*3 1649 | *n=*6 1899 | *n=*3 2483 | *n=*3 2508 | *n=*4 2882 | *n=*2 2509 |
| 6 | *n=*3 1657 | *n=*6 1637 | *n=*2 2130 | *n=*3 2023 | *n=*4 3291 | *n=*2 3401 |
| 8 | *n=*3 1262 | *n=*6 1466 | *n=*3 1813 | *n=*3 1739 | *n=*4 2794 | *n=*2 2305 |
| 24 | *n=*3 919.5 | *n=*6 624.5 | *n=*3 632.8 | *n=*3 459.2 | *n=*4 869.7 | *n=*2 929.3 |
| Note. Data are G_mean_, with a limit of quantification *=* 0.5 ng ml, 20 ng ml. G_mean_ calculated as exp [μ], where μ is the mean of the data on a log scale Abbreviations: G_mean_ = geometric mean; NC = not calculable | | | | | | |

| **Supplementary Table 4. Concentration and ratio of 4-β-hydroxycholesterol on Day 15 compared with Day 1 in the solid tumour trial** | | | | |
| --- | --- | --- | --- | --- |
| **Subject ID** | **Dose (mg)** | **Day 1 concentration (ng ml)** | **Day 15 concentration (ng ml)** | **Ratio  (Day 15/Day 1)** |
| E0001016 | 700 | 25.1 | 85.5 | 3.41 |
| E0003012 | 700 | 15.5 | 91.9 | 5.93 |
| E0003014 | 700 | 35.7 | 175 | 4.90 |
| E0001013 | 800 | 27.4 | 128 | 4.67 |
| E0002008 | 800 | 31.5 | 110 | 3.49 |
| G_mean_ (CV%) |  | 26.0 (32.8) | 114.1 (29.2) | 4.38 (23.9) |
| Abbreviations: CV = coefficient of variation; G_mean_ = geometric mean | | | | |

| **Supplementary Table 5: Best clinical response following AZD1208 treatment in the AML and solid tumour trials (clinical response analysis set)** | | | | | | |
| --- | --- | --- | --- | --- | --- | --- |
| Best response in AML study, *n*(%) | AZD1208  120 mg *n=*4 | AZD1208  240 mg *n=*6 | AZD1208  480 mg *n=*6 | – | AZD1208 700 mg *n=*7 | AZD1208  900 mg  *n=*9 |
| **Modified Cheson criteria** | | | | | | |
| CR | 0 | 0 | 0 | – | 0 | 0 |
| CRi | 0 | 0 | 0 | – | 0 | 0 |
| Morphologic leukaemia free | 0 | 0 | 0 | – | 0 | 0 |
| Partial remission | 0 | 0 | 0 | – | 0 | 0 |
| Non-response | 4 (100) | 6 (100) | 6 (100) | – | 7 (100) | 9 (100) |
| **Investigator assessed** | | | | | | |
| CR | 0 | 0 | 0 | – | 0 | 0 |
| CRi | 0 | 0 | 0 | – | 0 | 0 |
| Morphologic leukaemia free | 0 | 0 | 0 | – | 0 | 0 |
| Partial remission | 0 | 0 | 0 | – | 0 | 0 |
| Non-response | 3 (75.0) | 3 (50.0) | 3 (50.0) | – | 1 (14.3) | 2 (22.2) |
| **Best response in solid tumour study, *n* (%)** | **AZD1208  120 mg *n=*3** | **AZD1208 240 mg *n=*7** | **AZD1208  360 mg *n=*5** | **AZD1208  540 mg *n=*7** | **AZD1208 700 mg *n=*6** | **AZD1208 800 mg *n=*5** |
| **RECIST criteria** | | | | | | |
| CR | 0 | 0 | 0 | 0 | 0 | 0 |
| Partial response | 0 | 0 | 0 | 0 | 0 | 0 |
| Stable disease ≥6 weeks | 2 (66.7) | 5 (71.4) | 1 (20.0) | 3 (42.9) | 1 (16.7) | 1 (20.0) |
| Progression | 1 (33.3) | 2 (28.6) | 3 (60.0) | 3 (42.9) | 3 (50.0) | 3 (60.0) |
| Death | 0 | 0 | 0 | 0 | 1 (16.7) | 0 |
| Not evaluable | 0 | 0 | 1 (20.0) | 1 (14.3) | 2 (33.3) | 1 (20.0) |
| Abbreviations: AML = acute myeloid leukaemia; CR = complete response; Cri = complete response with incomplete recovery of blood counts; RECIST = Response Evaluation Criteria in Solid Tumours | | | | | | |

**Supplementary Figure 1. AML dose-escalation study geometric mean plasma concentration (± SD) of AZD1208 *vs* time by dose cohort for (A) Cycle 1, Day 1, and (B) Cycle 1, Day 14.**

Abbreviations: AML = acute myeloid leukaemia; SD = standard deviation.


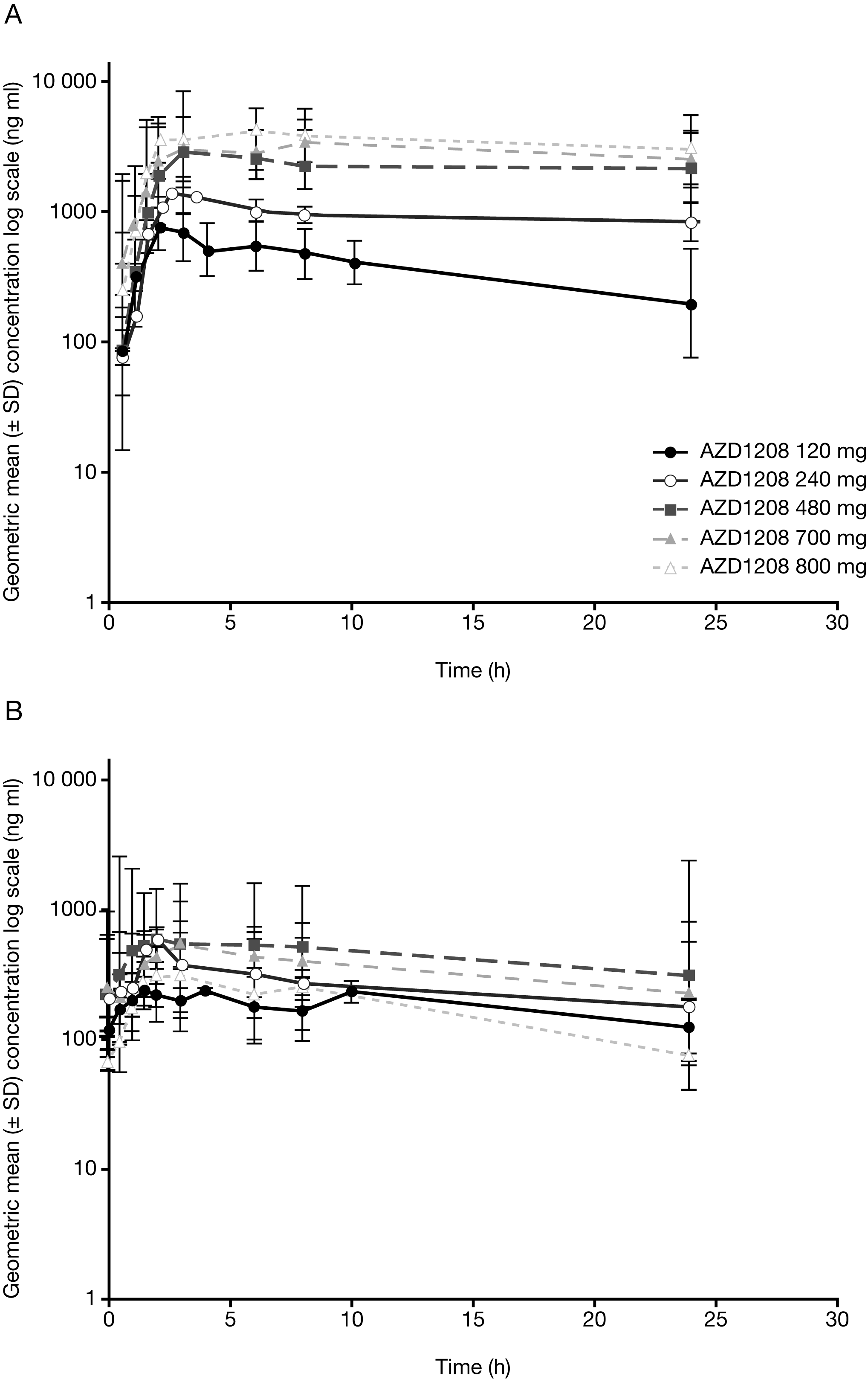


**Supplementary Figure 2. Solid tumour dose-escalation study individual and geometric mean values for C_max_ *vs* dose curve for (A) Cycle 0, Day 1, and (B) at steady state on Cycle 1, Day 15.**

Abbreviation: C_max_ = maximum plasma concentration.**
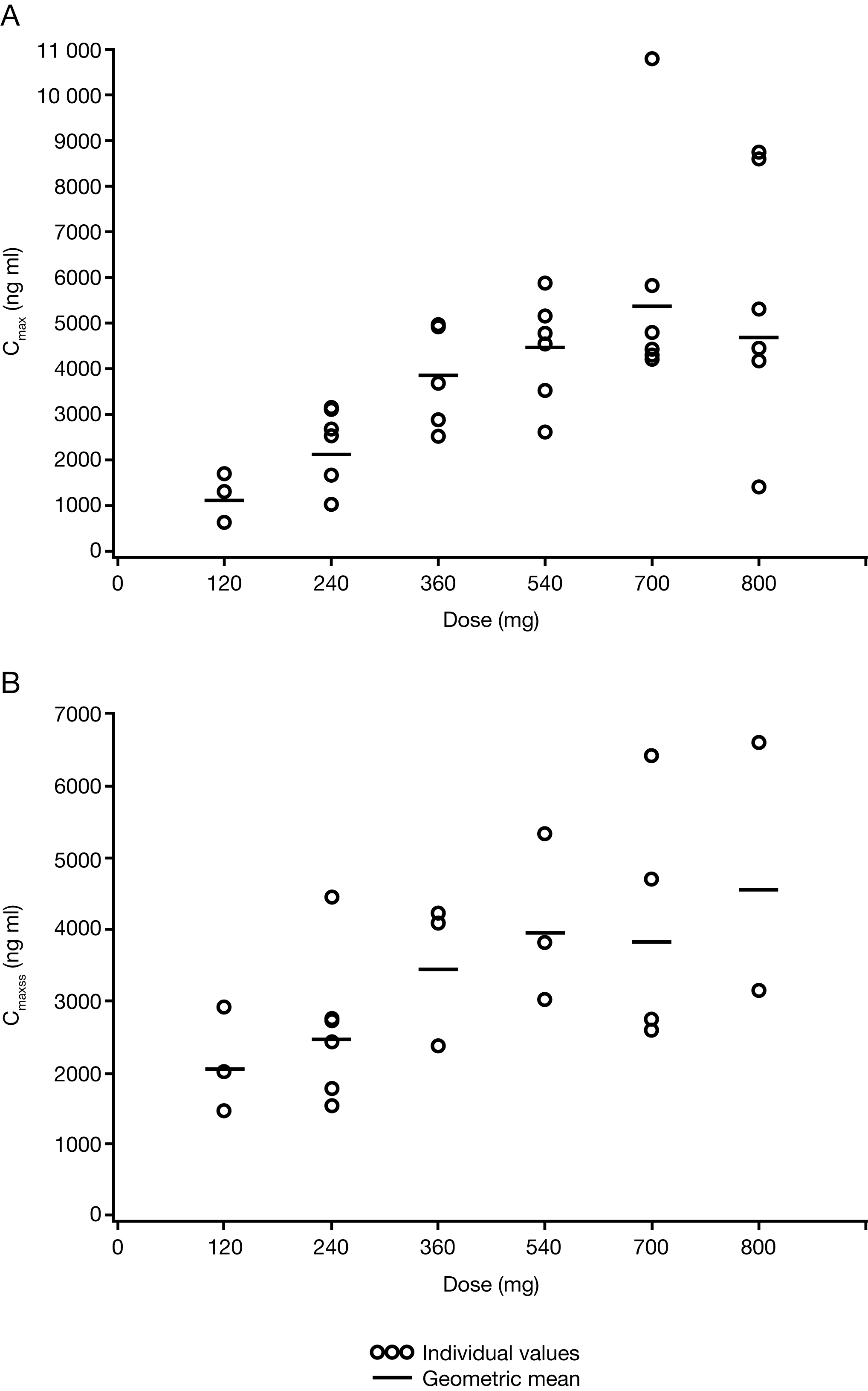
**

**Supplementary Figure 3. Solid tumour dose-escalation study individual and geometric mean values for AUC for (A) 0–24 h on Cycle 0, Day 1, and (B) 0 h to the end of treatment on Cycle 1, Day 15.**

Abbreviation: AUC = area under plasma concentration–time curve.**
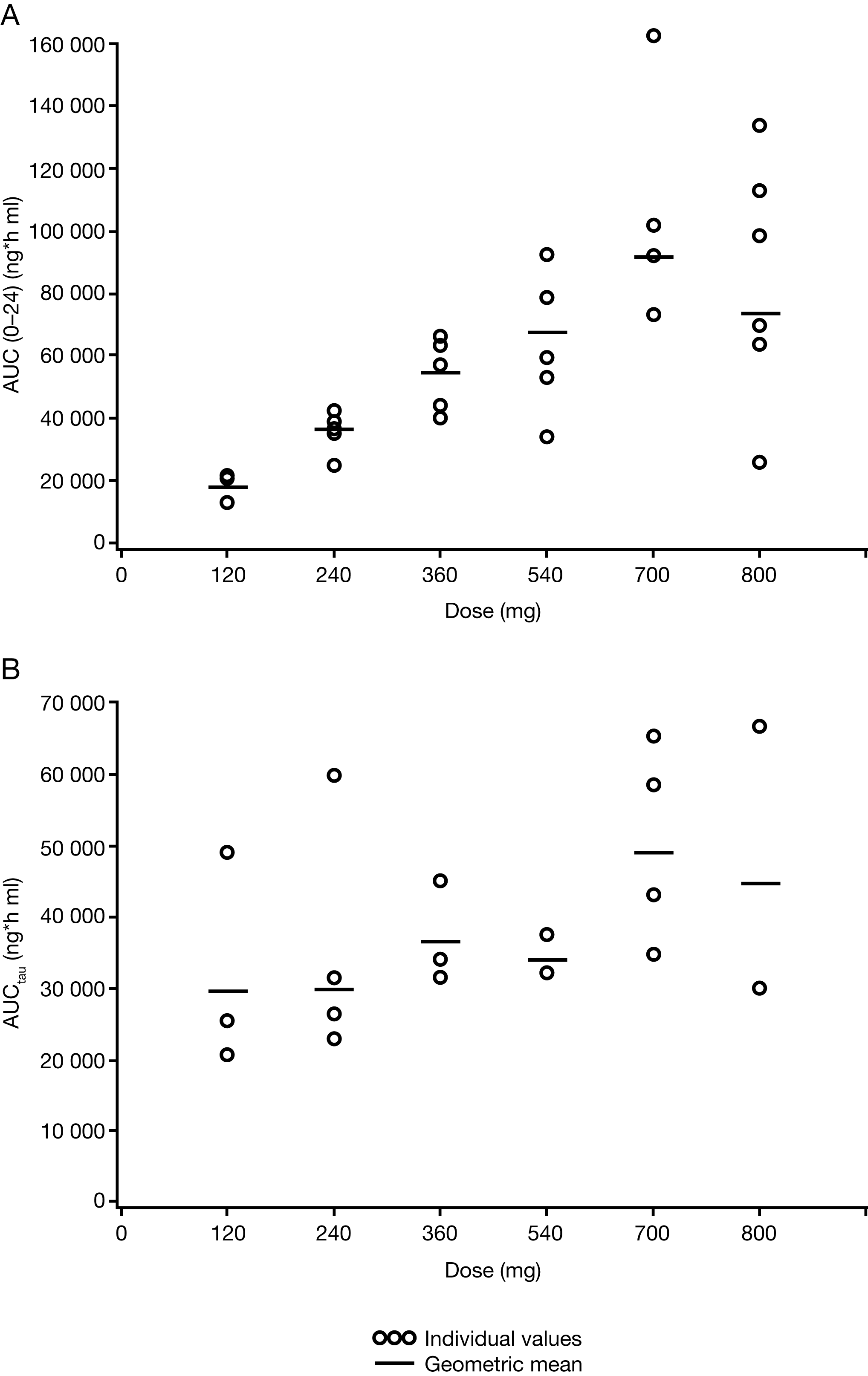
**

**Supplementary Figure 4. Measurement of cellular protein level changes by RPPA in AML blasts during therapy with AZD1208. Graphical representation of relative, normalised linear protein levels determined by RPPA of (A) 4E-BP1 S65, and (B) BAD S112.**

Cellular proteins were extracted from AML blasts from patients (*n=*6) isolated during therapy with AZD1208, and were analysed by RPPA. For all patients, samples were collected on Day 1. For the first two patients, the second set of samples were collected on Day 7 (dashed lines) and for the last four patients the second set of samples were collected on Day 14 (solid lines). One patient had an FLT3-ITD mutation and one patient had p53 mutation.

Abbreviations: AML = acute myeloid leukaemia; FLT3 = FMS-like tyrosine kinase 3; ITD = internal tandem duplication; mut = mutation; RPPA = reverse phase protein array.


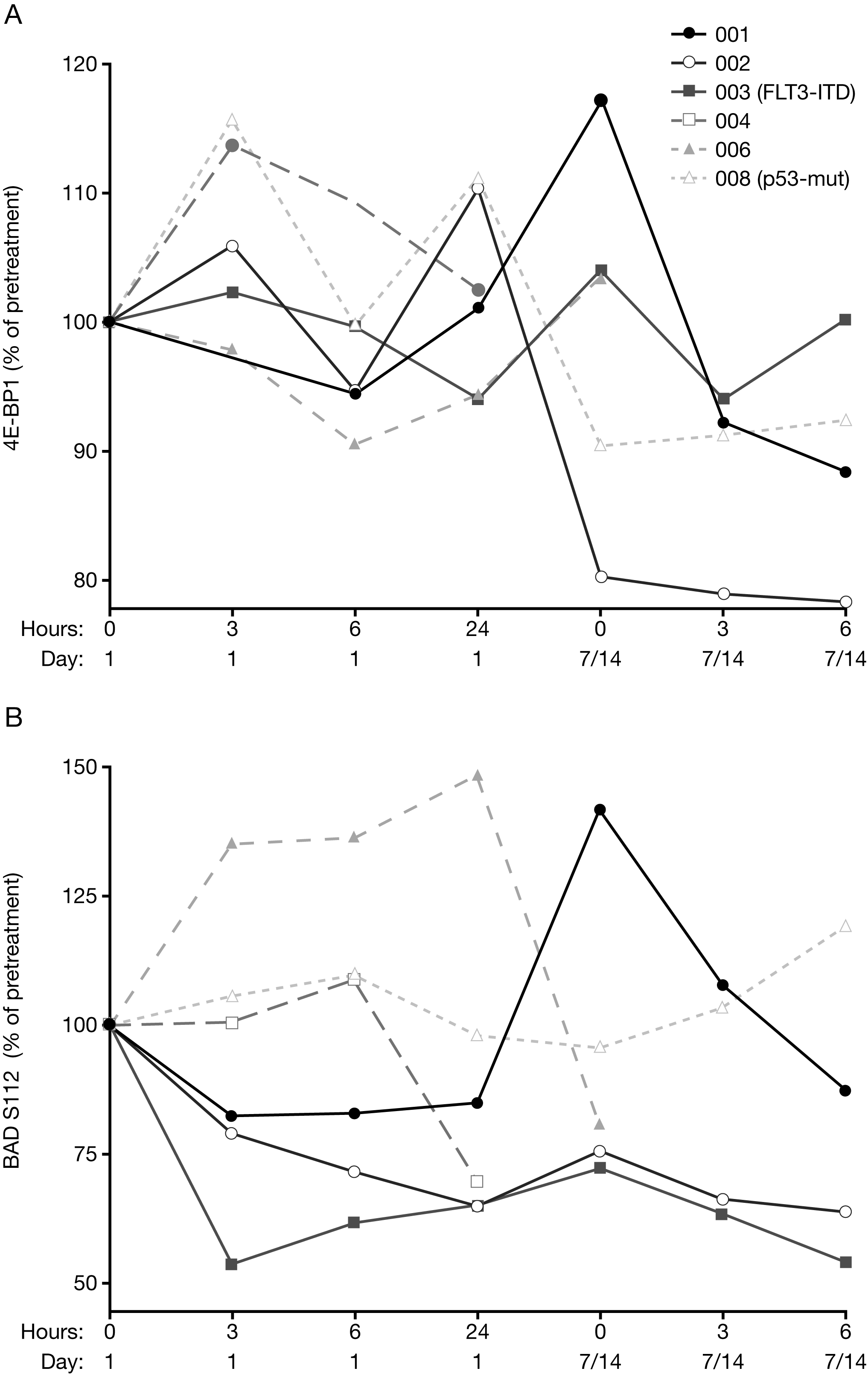


**Supplementary Figure 5. (a) RPPA heatmap of modulated protein levels in AML blasts during therapy with AZD1208. Cellular proteins were extracted from AML blasts from patients (*n=*6) isolated during therapy with AZD1208 and were analysed by RPPA. Graphical representation of changes in phosphorylated protein levels are shown in (B) 4E-BP1 T37/46, (C) PRAS40 T246 and (D) mTOR S2448.**

The heatmap was generated using unsupervised clustering analysis.
For all patients, samples were collected on Day 1.
For the first two patients, the second set of samples were collected on
Day 7 (dashed lines) and for the last four patients the second set of samples were collected on Day 14 (solid lines).
One patient had an FLT3-ITD mutation and one patient had p53 mutation.

Abbreviations: AML = acute myeloid leukaemia; FLT3 = FMS-like tyrosine kinase 3; ITD = internal tandem duplication; RPPA = reverse phase protein array.


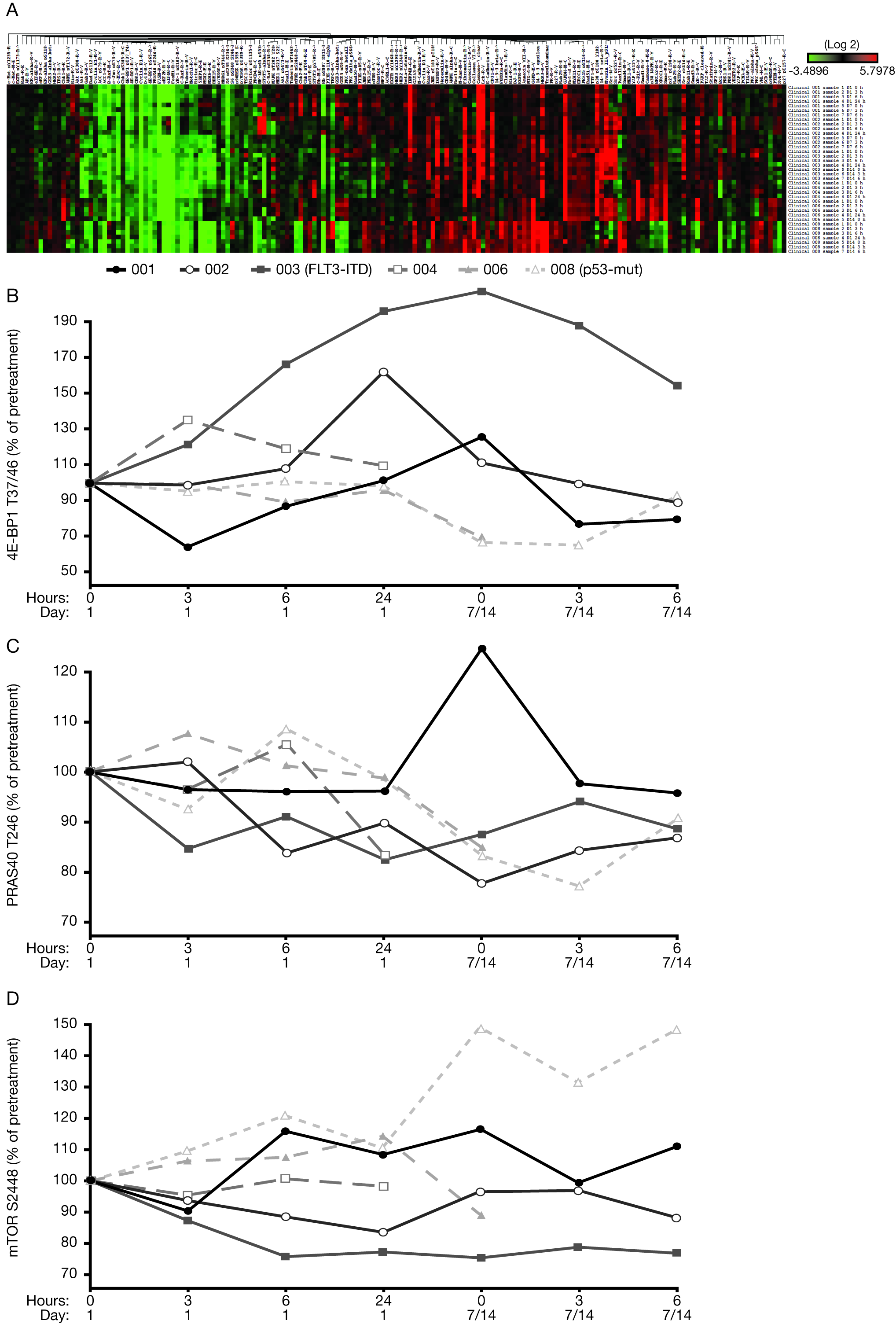

Supplement: Supplementary file 1 — Study 1 and 5 supplementary appendix [file 41416_2018_82_MOESM1_ESM.docx]
