# Supplementary figures and images for: Phase I studies of AZD1208, a proviral integration Moloney virus kinase inhibitor in solid and haematological cancers
Source: Br J Cancer. 2018 May 16;118(11):1425–33. doi: 10.1038/s41416-018-0082-1 (PMC5988656; doi:10.1038/s41416-018-0082-1)

**A**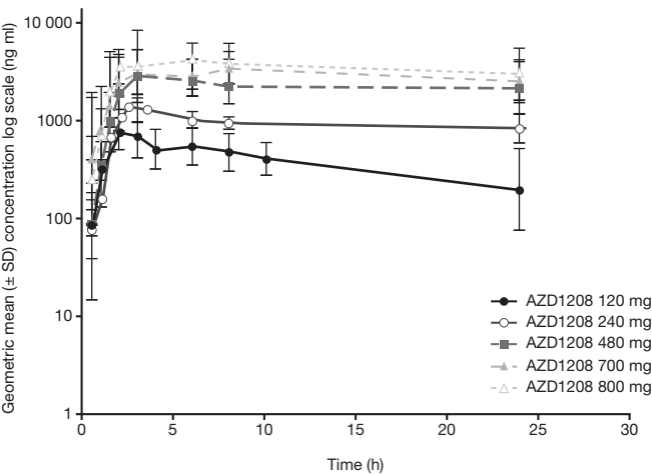**B**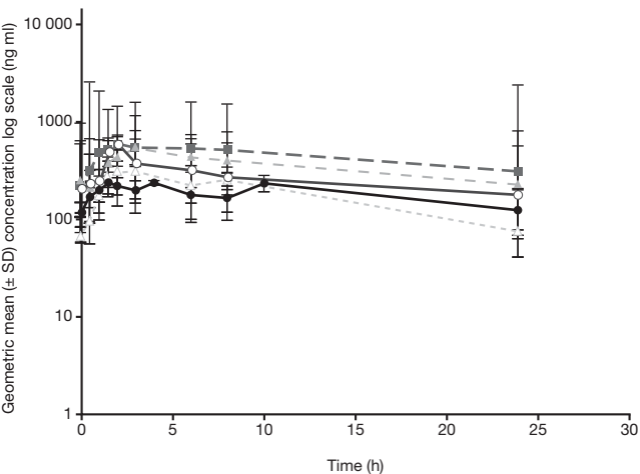

Supplement: Supplementary file 2 — Supplementary Figure 1 [file 41416_2018_82_MOESM2_ESM.pdf]

**A**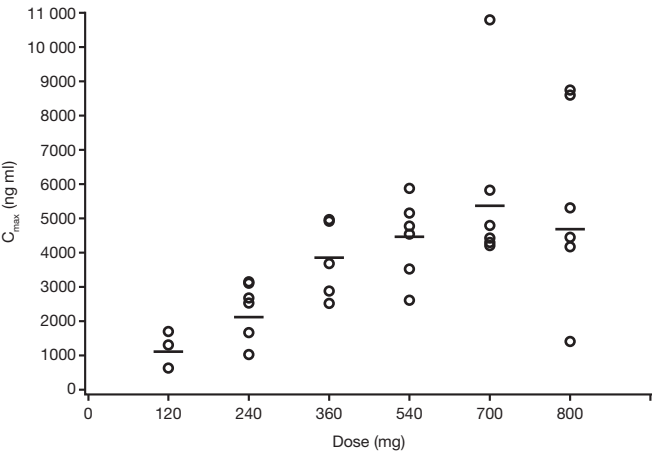**B**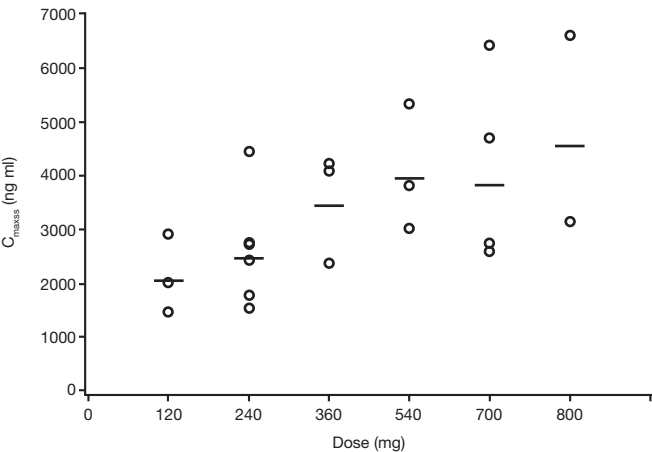

ooo Individual values

— Geometric mean

Supplement: Supplementary file 3 — Supplementary Figure 2 [file 41416_2018_82_MOESM3_ESM.pdf]

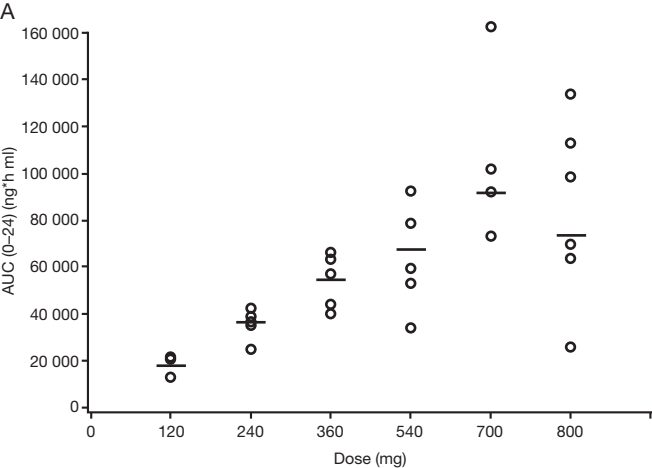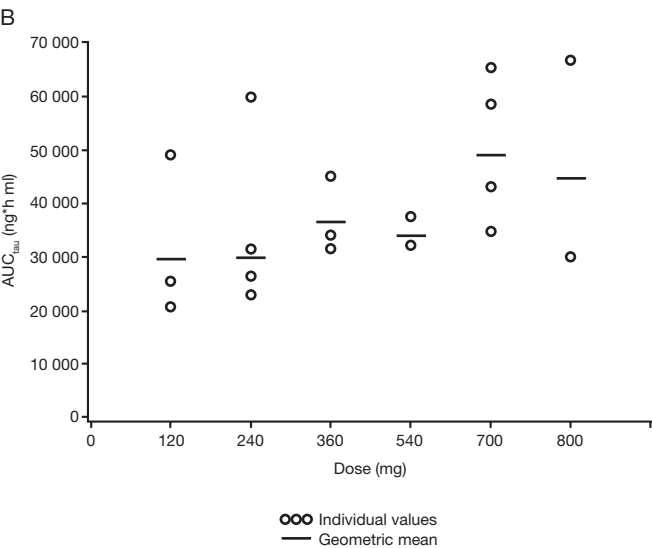

Supplement: Supplementary file 4 — Supplementary Figure 3 [file 41416_2018_82_MOESM4_ESM.pdf]

**A**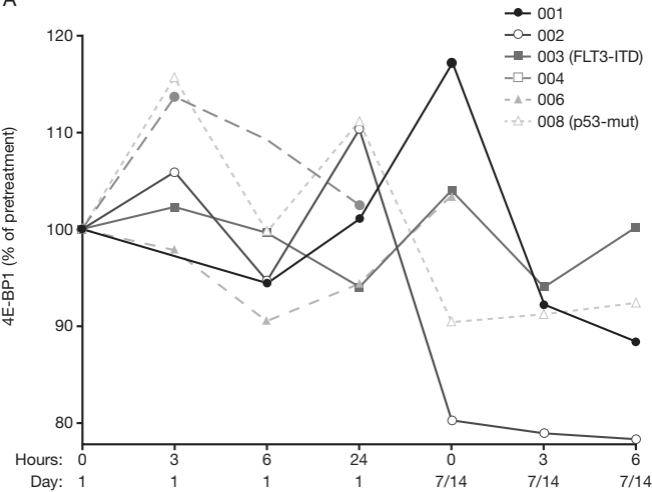**B**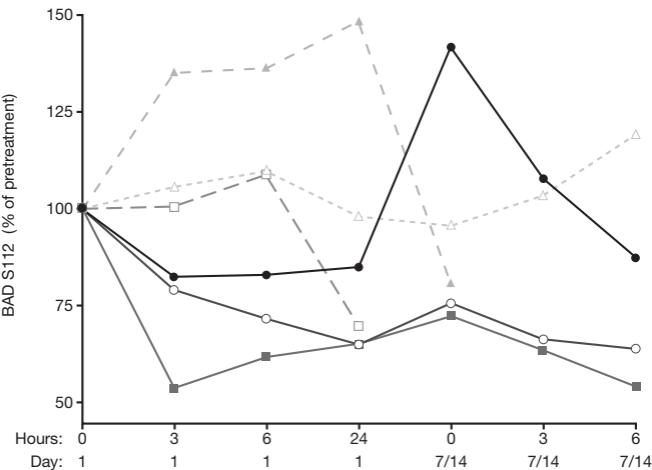

Supplement: Supplementary file 5 — Supplementary Figure 4 [file 41416_2018_82_MOESM5_ESM.pdf]

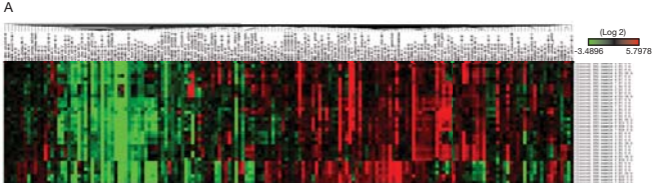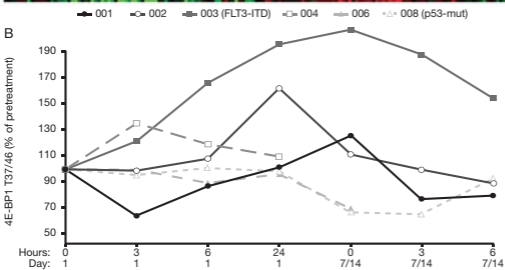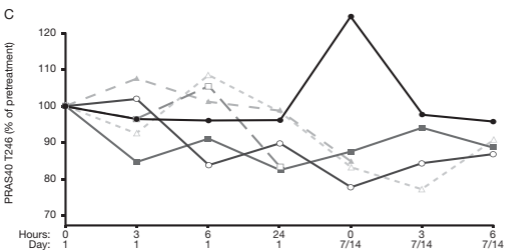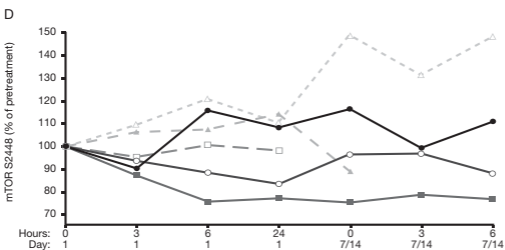

Supplement: Supplementary file 6 — Supplementary Figure 5 [file 41416_2018_82_MOESM6_ESM.pdf]
